# Green remediation of 4-nitrophenol-contaminated industrial wastewater using biochar–metal composites: a review

**DOI:** 10.1039/d6ra05330k

**Published:** 2026-09-01

**Authors:** Abraham Stuvart, Arun Thesingu Rajan, Hemanth P. K. Sudhani, Natarajan Raman

**Affiliations:** a School of Sciences, Division of Chemistry, SRM Institute of Science and Technology Tiruchirappalli 621105 Tamil Nadu India arunt@srmist.edu.in; b School of Biosciences, Department of Biotechnology, SRM Institute of Science and Technology Tiruchirappalli 621105 Tamil Nadu India sudhanip@srmist.edu.in; c Science Instrumentation Centre, Ayya Nadar Janaki Ammal College (Autonomous) Sivakasi-626124 Tamil Nadu India

## Abstract

The contamination of water bodies by industrial effluents is a growing global concern, especially due to the presence of persistent and bio-accumulative pollutants such as 4-nitrophenol. 4-Nitrophenol is extensively used in pharmaceutical, insecticide, dye and leather industries, resulting in adverse effects on human health and environment. Conventional treatment methods such as adsorption, advanced oxidation, biodegradation and electrochemical reduction have numerous limitations, including incomplete mineralization, elevated operational costs, the generation of secondary pollutants, and challenges related to scalability. Recent studies have shown that biochar–metal composites are promising alternatives, which possess the advantages of the char's physicochemical properties combined with the catalytic activity of metal nanoparticles. The aim of this review is to assess the available literature on the production, adsorption and catalytic properties of biochar–metal composites as well as their application in 4-NP remediation. The influence of feedstock, pyrolysis conditions and metal incorporation on the formation of biochar composites is discussed in detail. This review highlights the importance of life cycle assessment and economic analysis, which can provide an understanding of the potential environmental and economic implications of biochar composites. However, there are several challenges associated with material strength, size constraints, metal leaching potential and commercial availability. This review is an attempt to connect laboratory-scale research with real-world implementation by outlining the important knowledge gaps and proposing future research including integrated studies, novel preparation techniques, mechanistic investigations and policy development. Hence, biochar–metal composites can enhance the conventional water treatment methods and provide effective solutions for mitigating global environmental contamination caused by pollutants like 4-nitrophenol.

## Abstract 2

Biochar–metal composites enhance the removal of 4-nitrophenol from contaminated water through improved adsorption and catalytic activity, offers an efficient and sustainable strategy for pollutant removal and environmental remediation.

## Graphical abstract

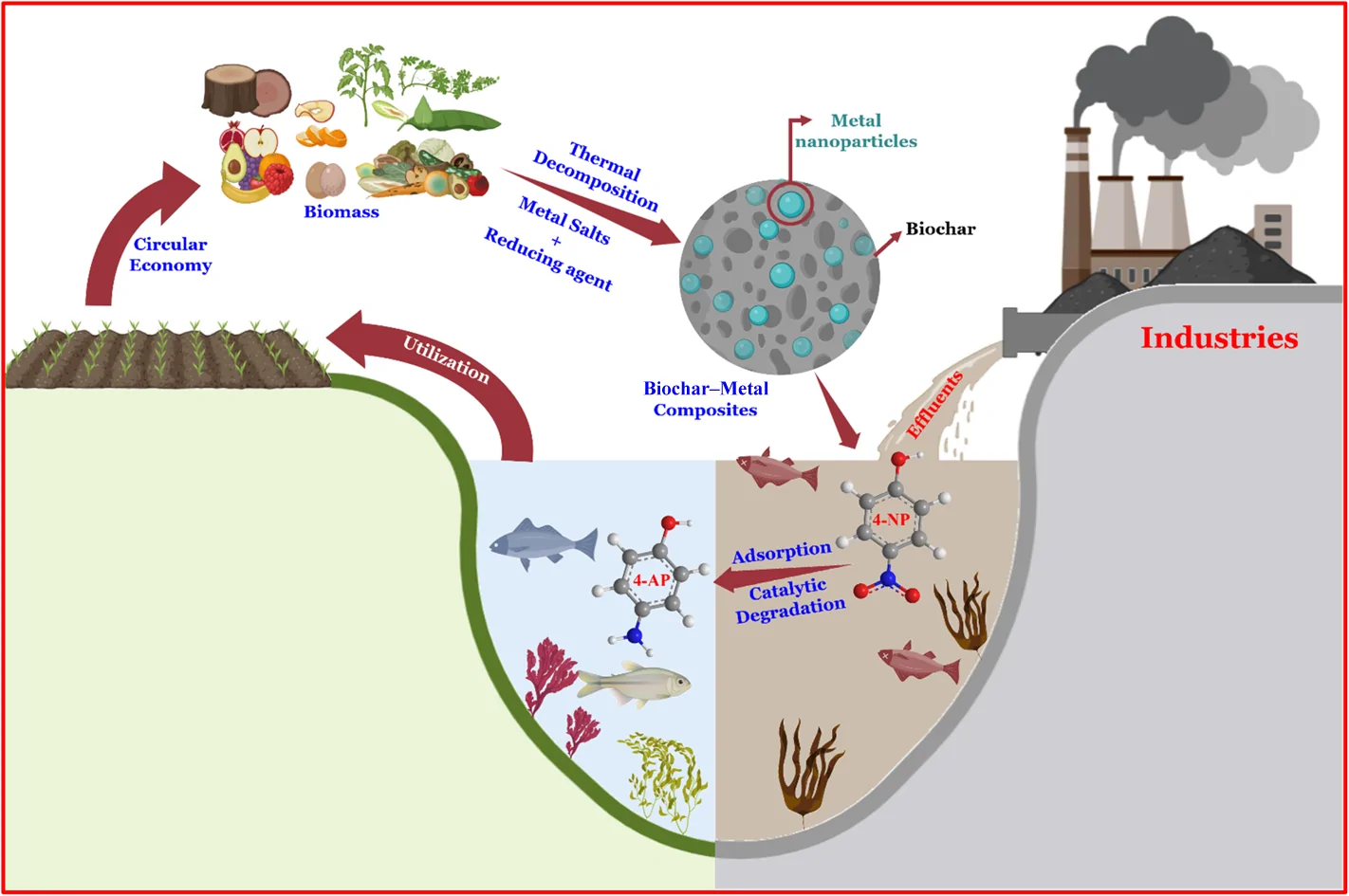

## 1 Introduction

### 1.1 Environmental significance of water pollution

Water pollution constitutes a significant environmental challenge of the 21st century, principally resulting from the increased release of industrial contaminants into waterbodies. Freshwater is very important but scarce, comprising about 2.5% of the total water on the surface of the planet.^1^ The rate of industrial activities and the pace of urbanization are increasing rapidly, and they both contribute to water pollution, posing risks to human health and ecological systems. Industrial effluents are frequently either untreated or poorly treated, consequently introducing organic and inorganic contaminants into water bodies.^2–4^ Pollutants such as pharmaceuticals, pesticides, dyes, phenolic compounds and heavy metals, which are often found in industrial effluents, resist natural degradation, hence hindering ecosystem recovery and affecting human health.^5^ As a result, ensuring the accessibility of safe and clean water sources has emerged as a significant global issue, necessitating the exploration of innovative and environmentally sustainable methods for water purification.^6^

Nitrophenols are particularly concerning pollutants due to their high toxicity, potential for bioaccumulation and persistence in aquatic ecosystems. These substances, frequently discharged by pharmaceutical, pesticide, dye and petrochemical industries, are designated as priority pollutants by the United States Environmental Protection Agency (USEPA) and the European Union Water Framework Directive (EUWFD).^7–9^

**Fig. 1:**
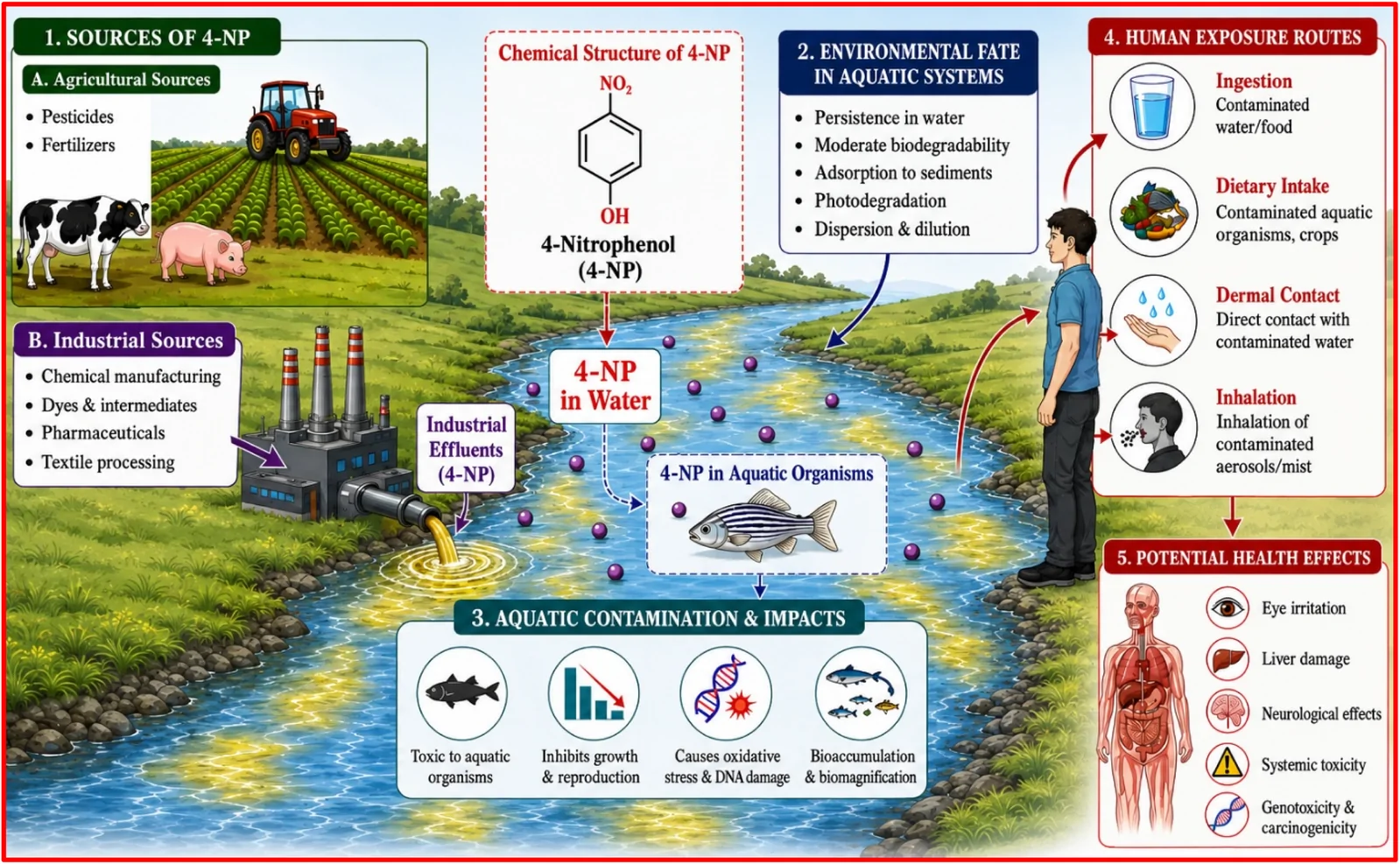
Industrial sources of 4-NP contamination, and the environmental fate and associated health effects of 4-NP.

Despite firm regulations, conventional wastewater treatment facilities fail to entirely eliminate nitrophenols, resulting in their accumulation in surface and groundwater sources. The persistent characteristics of these pollutants need the formulation of effective, sustainable, and economical remediation strategies. Among the several nitrophenol derivatives, 4-nitrophenol (4-NP) has drawn significant attention due to its extensive industrial applications and severe environmental footprint.^10,11^Fig. 1 shows the industrial sources of 4-NP contamination, its environmental fate, and associated health effects.

### 1.2 4-Nitrophenol (4-NP): a harmful pollutant

#### 1.2.1 4-Nitrophenol: chemical structure, sources, and environmental fate

**Fig. 2:**
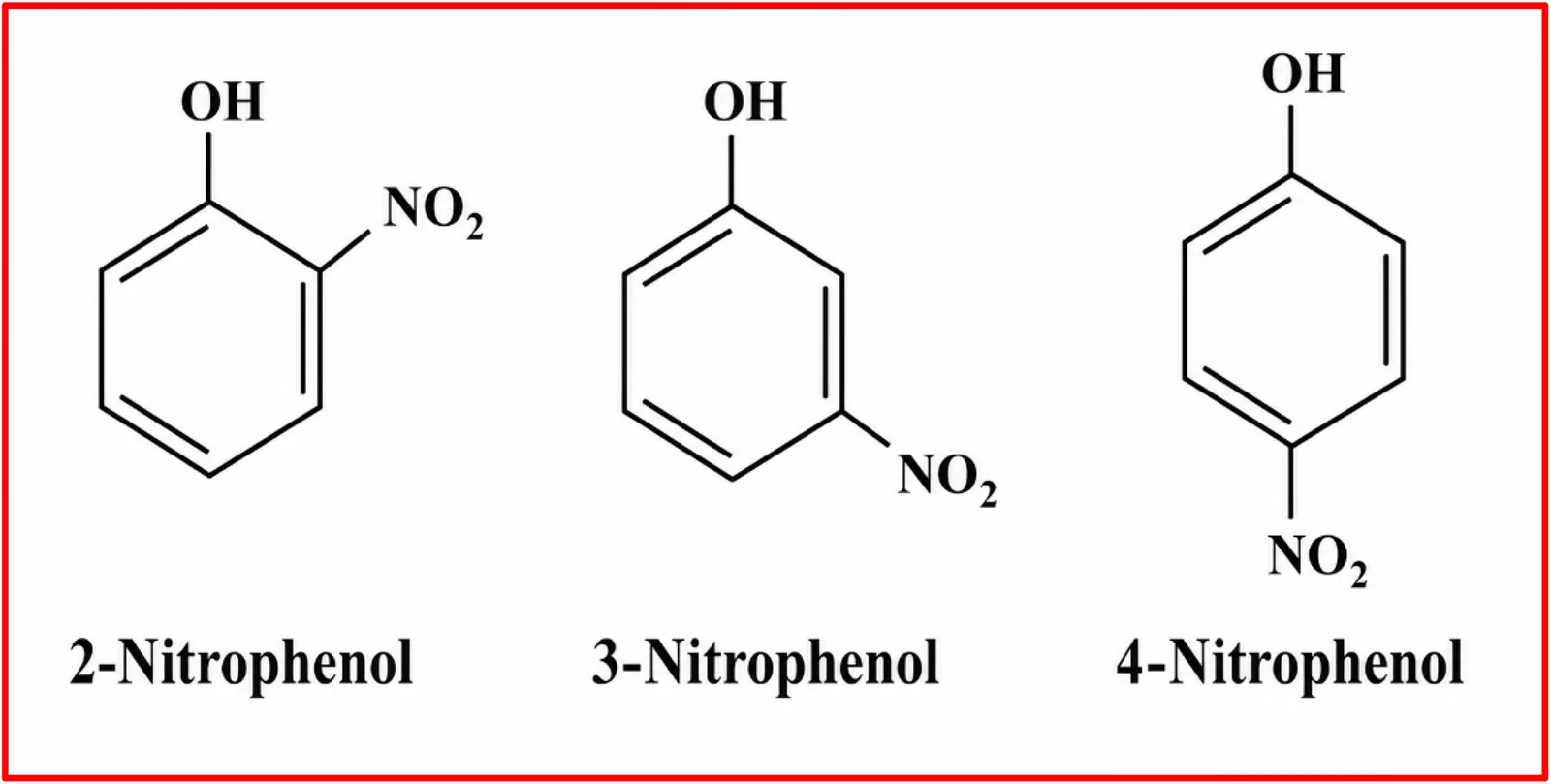
Isomeric forms of nitrophenols.

Nitrophenols include a series of chemicals whose molecular structures have a nitro group (–NO_2_) at the phenol ring. Among them, 4-nitrophenol (4-NP) has garnered notable attention due to its widespread usage in industries.^12^ 4-NP is an aromatic nitro compound that is structurally simple but poses considerable environmental toxicity. It is widely employed across different industries, even with its considerable environmental risks.^13^ The molecular structure of 4-NP features a benzene ring substituted with a hydroxyl (–OH) group and a nitro (–NO_2_) group, leading to significant stability and moderate solubility in water.^14^ The nitro group significantly withdraws electrons away from the aromatic ring, resulting in an electron-deficient environment that improves its stability and makes it more resistant to biodegradation.^15^ The isomeric forms of nitrophenol are shown in Fig. 2. The electronic configuration also renders 4-NP resistant to microbial degradation and susceptible to redox changes, which contributes to its persistence in aquatic environments. The physicochemical profile of 4-NP, marked by moderate aqueous solubility, low volatility, and the formation of pale yellow crystalline solids, contributes to its environmental persistence.^16^

#### 1.2.2 Sources and environmental fate

4-NP functions as a vital intermediate compound in various industrial sectors, such as pharmaceuticals, agrochemicals, dyes, and leather processing.^17^ 4-NP plays a significant role in the synthesis of analgesics, including paracetamol, which is extensively utilised worldwide for pain relief.^18^ The significant industrial use leads to the unintentional discharge of 4-NP into aquatic ecosystems *via* untreated or poorly treated effluents.^19^ Industrial discharges, especially from pharmaceutical manufacturing, pesticide production and leather industries, often contain residual 4-NP, resulting in considerable environmental contamination.^20^

**Fig. 3:**
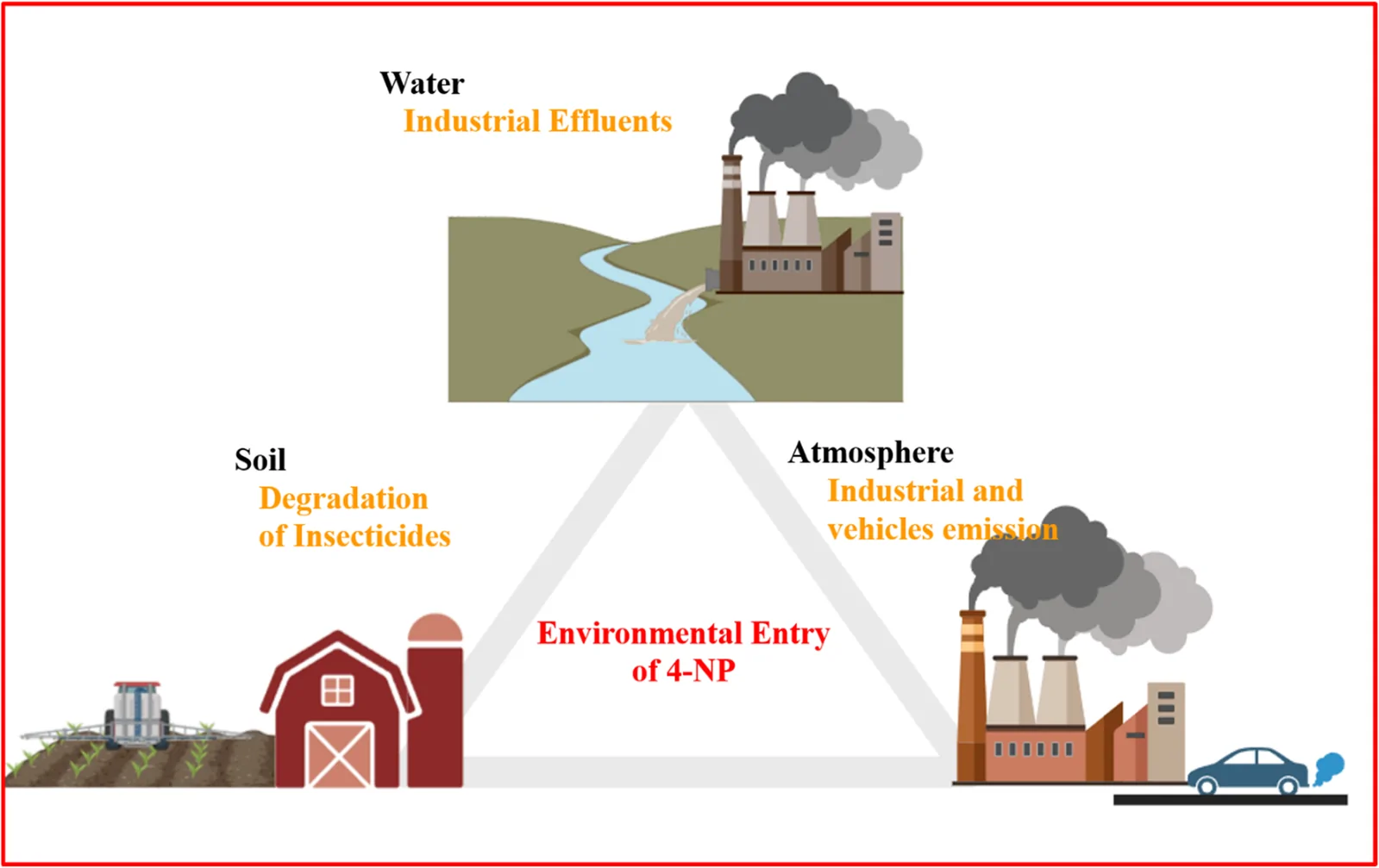
Environmental entry pathways of 4-NP.

4-NP exhibits persistence in aquatic environments owing to its chemical stability, low photodegradation, and restricted biodegradability. The compound accumulates in sediments and aquatic environments, gradually bioaccumulating in organisms and potentially biomagnifying within food chains.^21^ The resilience of natural attenuation increases environmental exposure risks, impacting aquatic biodiversity and human populations dependent on contaminated water resources. Fig. 3 illustrates the environmental entry pathways of 4-NP.

#### 1.2.3 Health and ecological risks

**Fig. 4:**
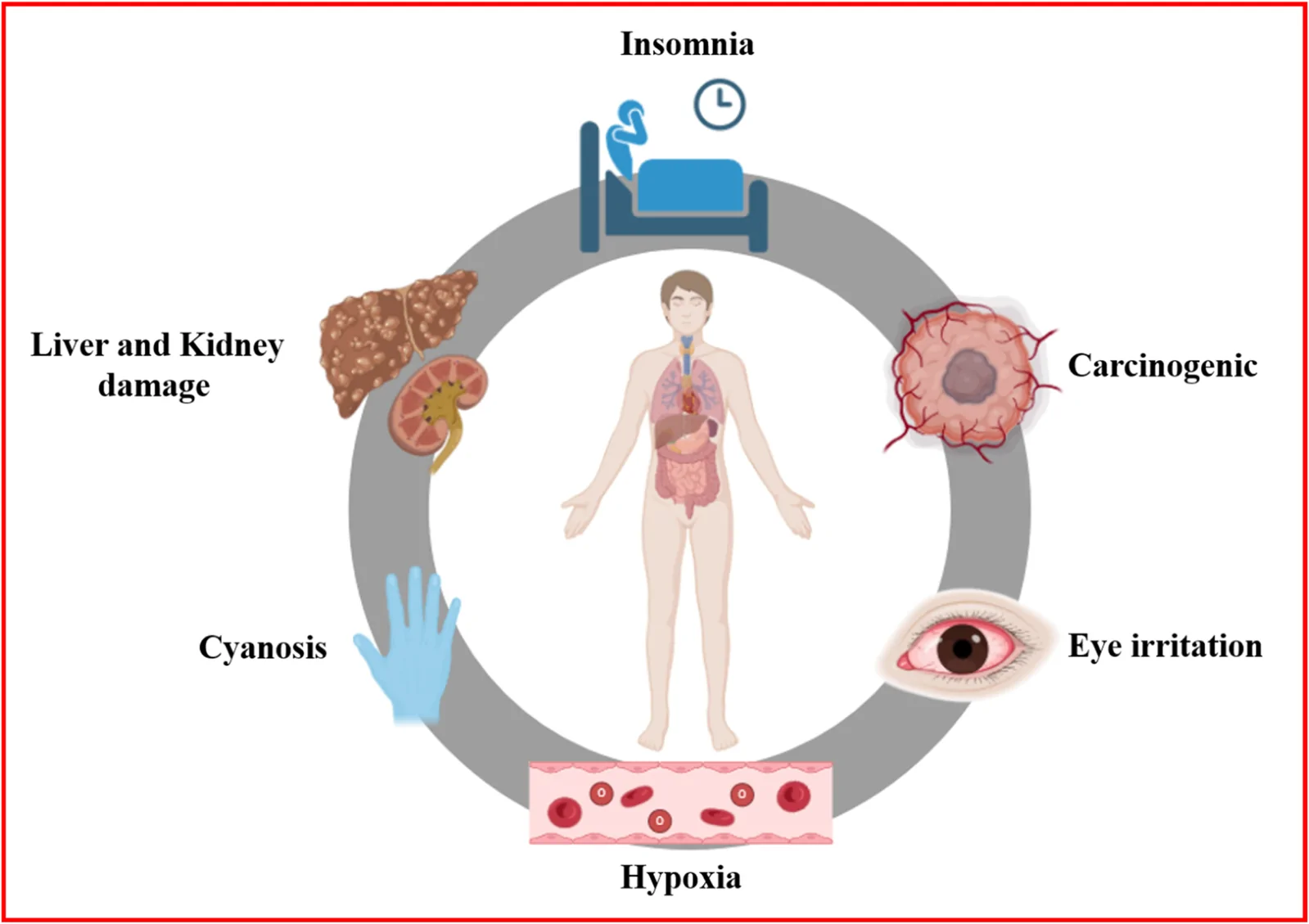
Impact of 4-NP on human beings.

4-NP poses significant ecological and health hazards owing to its toxic nature, stability, and tendency for bioaccumulation. It disrupts aquatic biodiversity by impacting microbial communities, hindering enzymatic activities and impairing photosynthetic processes in aquatic plants. The bioaccumulation across trophic levels exacerbates these harmful effects, endangering biodiversity and ecological balance. Fig. 4 illustrates the impact of 4-NP on human beings.

Additionally, human exposure to 4-NP primarily occurs *via* contaminated water or occupational settings. Acute human exposure may lead to significant symptoms, including cyanosis due to methemoglobinemia, headaches, respiratory distress, nausea, and severe irritation of the eyes and mucous membranes.^22^ Chronic exposure poses significant risks, including endocrine disruption, liver and kidney dysfunction, neurological complications, and elevated cancer risks.^23,24^ The negative health effects linked to 4-NP highlight the critical necessity for effective and sustainable remediation technologies.

### 1.3 Current remediation technologies and limitations

Current remediation technologies for 4-NP primarily include adsorption, chemical oxidation, photocatalysis, biological degradation and electrochemical methods. Adsorption using activated carbon is effective yet non-destructive, requiring secondary treatment or regeneration.^25^ Chemical oxidation processes, such as Fenton's oxidation and ozonation, effectively degrade 4-nitrophenol. However, they frequently generate harmful intermediates and necessitate significant energy inputs.^26^ Photocatalytic methods demonstrate efficiency in laboratory settings. However, they encounter practical challenges related to scalability, catalyst stability, and sensitivity to environmental factors.^27^

Biological treatments are environmentally advantageous but exhibit limited effectiveness in industrial settings due to slow reaction kinetics, incomplete degradation, and susceptibility to inhibitory substances.^28^ Electrochemical methods demonstrate significant remediation efficiency.^29^ However, they are associated with high costs, energy-intensive processes, and complexities in large-scale implementation. These constraints require the investigation for sustainable and cost-effective remediation strategies that effectively remove 4-NP from water while also considering the lifecycle impacts of the treatment process.^30^ Biochar–metal composites represent a viable solution, characterized by cost-effective production from waste biomass and potential for carbon sequestration, alongside high efficiency in pollutant removal.^31,32^ Nonetheless, gaps persist in the comprehensive understanding and optimization of these materials for the remediation of 4-NP.

### 1.4 Research gaps, importance, and objectives

The review fills several knowledge gaps in the application of biochar and its composites for 4-NP degradation. Although studies exist on biochar for pollutant degradation, a systematic review of 4-NP remediation is lacking. Previous reviews of nitrophenols have been directed towards analytical methods or pre-treatment techniques, while reviews of biochar composites frequently highlight soil applications or heavy metals instead of nitroaromatic pollutants. There are still many questions that remain unanswered regarding the comparison of different biochar composite preparation methods in terms of sustainability and effectiveness for 4-NP removal.

The fundamental mechanism of adsorption and catalytic degradation of these materials is still unknown. Besides, the life-cycle environmental impact, reusability, and scalability of these materials have not been fully investigated.

In this review article, the following critical questions are proposed to address the existing gaps: (i) what are the limitations of current 4-NP remediation methods and how can biochar–metal composites solve these limitations? (ii) what impact does different biochar production techniques (such as pyrolysis and hydrothermal) have on the properties that are relevant to 4-NP adsorption and which one of these methods is most sustainable and commercially viable? (iii) how does the application of metal–biochar composites enhance the removal of 4-NP (adsorptively or catalytically) compared to the pure biochar and what insights can be learned about the various metal additives? (iv) What are the mechanistic pathways of adsorption and catalytic degradation of 4-NP on these composites as revealed by advanced characterization, kinetics, and computational modelling? (v) What are some preliminary life cycle assessments, energy analyses, and cost-benefit assessments of using biochar composites for water treatment and what challenges remain for their scale-up? This review is an attempt to contribute to the development of the field of 4-NP remediation using biochar by a critical and comparative analysis of the previous research studies, determining research gaps and proposing further directions. Importantly, rather than having a list of previous research studies, we evaluate the methods used in these studies and the conclusions drawn from them to determine what approaches are most effective and why, and where there are inconsistencies or trade-offs. To this end, it is our hope that this approach will provide a clearer roadmap to researchers and practitioners in developing the next generation of sustainable remediation technologies for nitrophenols.

## 2 Biochar production methods

**Fig. 5:**
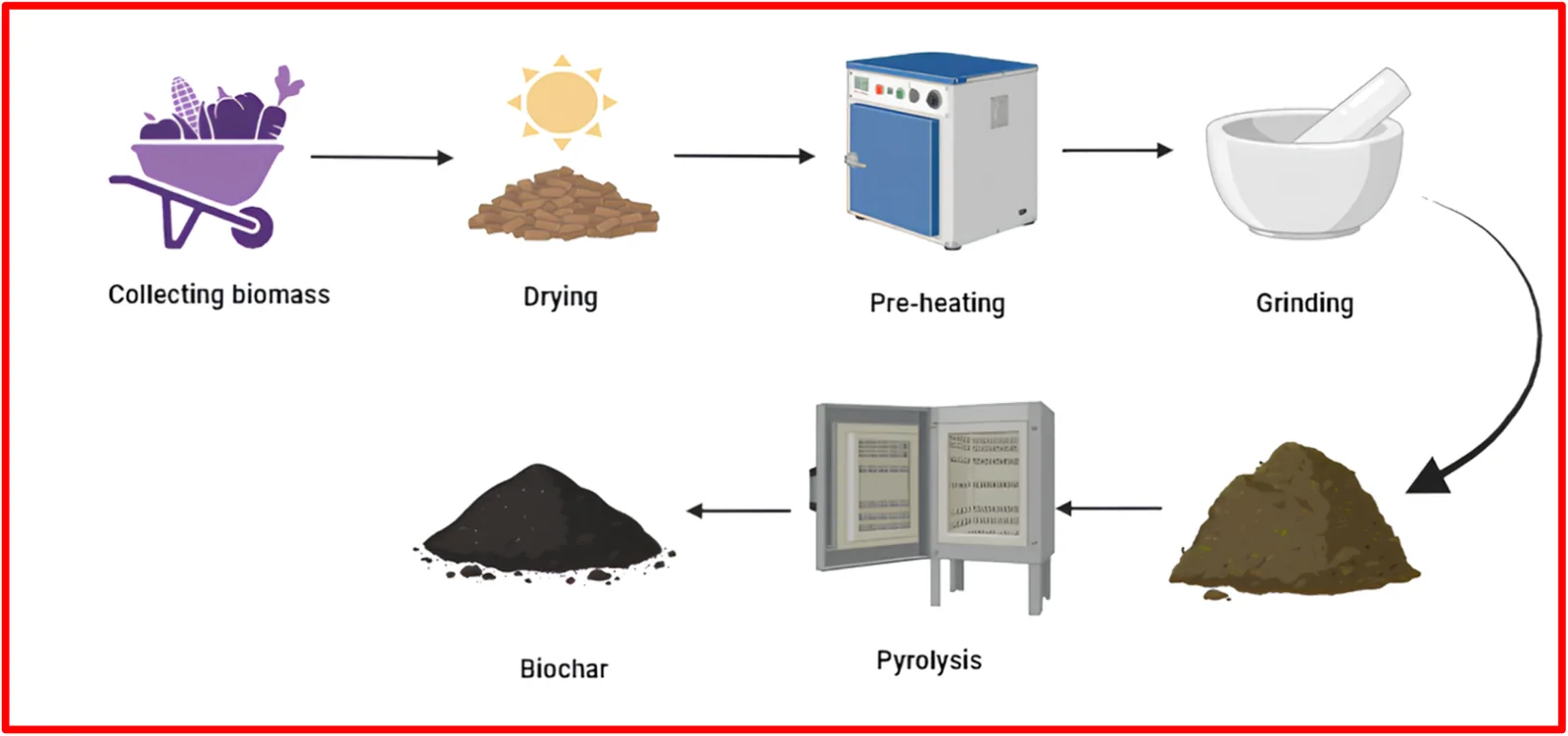
Preparation and processing of biomass-derived biochar.

Biochar production is a complex process that involves several steps and the first one is the choice of feedstock, which is a biomass, and the feedstock can be any organic matter including wood, agricultural residues or even certain types of wastes. Biomass choice is important because it determines the qualities of the produced biochar, and therefore, the biomass should be chosen depending on the intended use as a soil conditioner, carbon storage, or otherwise.^33^ When the biomass has been chosen, it is gathered and then conditioned to remove moisture. Drying is a significant stage because the presence of moisture at this stage influences the efficiency of the next stage,^33^ that is, pyrolysis. It may also be done to eliminate residual moisture and other gases that may hinder the process of pyrolysis. The biomass is then exposed to pyrolysis which is the process of thermal decomposition that is done under an oxygen or inert atmosphere, so as to guarantee that the organic matter undergoes a process of converting to biochar instead of burning to ash.^34^Fig. 5 depicts the preparation and processing of biomass-derived biochar.

### 2.1 Pyrolysis

Pyrolysis is a thermochemical process that decomposes biomass in the absence of oxygen. Biomass pyrolysis generally occurs at temperatures ranging from 300 to 1000 °C.^35^ The biomass feedstocks used for pyrolysis are mainly obtained from wood, agricultural residues, kitchen wastes and other biomass-derived sources. This process converts biomass into three main products: solid (biochar), liquid (bio-oil) and gaseous product (Syn-gas). The pyrolysis of biomass undergoes three stages: (1) drying phase, where moisture in the biomass is vaporized below 200 °C without any chemical changes; (2) devolatilization, where biomass breaks down chemically above 200 °C, producing volatile gases, liquid bio-oil and small amount of solid residues; and (3) char formation, where the remaining materials become carbon-rich biochars after most volatile compounds are driven off. The final composition of the biochar largely depends on the type of feedstock used.^36^ Pyrolysis can be classified into different types based on the operating temperature, heating rate and residence time.

#### 2.1.1 Slow pyrolysis

Slow pyrolysis operates at a slow heating rate 10 °C min^−1^ and relatively low temperatures, usually around 400–750 °C.^37^ This process enhances the decomposition of biomass during an extended residence time. It facilitates maximum conversion of biomass into biochars and bio-oils, with yields ranging from 50 to 60 wt% and 80 to 90 wt%, respectively.^38,39^ The Hazelnut shell (HZS) and spent coffee ground (SCG) biochars were produced through pyrolysis at temperatures of 450 °C and 550 °C. The impact of pyrolysis temperature on biochar yield and morphology is significant. Increasing the temperature enhances the reaction rates but slightly reduces the biochar yield (17.7–23.1 wt% for SCG and 18.9–21.0% wt% for HZS), while favoring bio-oil and gas production due to secondary volatile cracking. Fig. S1 shows distinct morphologies for SCG (uniform particles of 5–10 µm forming aggregates) and HZS (irregular, fragmented particles of 5–50 µm), with particle size reducing after pyrolysis at 450 °C due to carbonization. At 550 °C, large pores are evident, with HZS_550 exhibiting a more homogeneous structure attributed to its higher fixed carbon content.^40^

#### 2.1.2 Fast pyrolysis

In fast pyrolysis, the biomass is rapidly heated to high temperatures; the heating rate is 700 °C s^−1^ and higher temperatures are typically around 400–650 °C.^41^ The process maximizes bio-oil production typically yielding 70% to 75%, while biochar yields are lower generally around 12%.^42,43^ Fast pyrolysis is particularly useful for producing liquid fuels. Mallee wood was subjected to both slow and fast pyrolysis at 500 °C. Biochar yields of the two processes were 20.16% and 16.23%, respectively. This indicates that employing fast pyrolysis instead of slow pyrolysis will reduce the yield of the biochar.^44^ The biochar prepared from banana pseudo-stem by fast pyrolysis had a lower carbon content than that prepared by slow pyrolysis. The surface area of biochar prepared by two methods was 0.638 m^2^ g^−1^ and 1.025 m^2^ g^−1^. Additionally, the biochar produced by slow pyrolysis had a high number of pores than the biochar produced from fast pyrolysis. This demonstrates that the biochar produced by the fast pyrolysis has lower fixed carbon content and surface area and less number of pores than that produced by slow pyrolysis.^45^

#### 2.1.3 Flash pyrolysis

Flash pyrolysis is an even more rapid process; the biomass is treated at a heating rate >1000 °C s^−1^, temperatures are often 1000 °C, and the residence time is extremely short, approximately 0.5 to 3 seconds. Like fast pyrolysis, flash pyrolysis rapidly vaporizes the biomass at high temperatures, predominantly forming bio-oil and gaseous products. The extremely short residence time and high operating temperature minimize the formation of solid residues, thereby leaving only a small amount of biochar behind.^46,47^ The bio-oil yield is 75% and the biochar yield is below 10% in this process.^48^ The yield of biochar produced from maize-cob by flash pyrolysis was lower than that of the other two pyrolysis methods, and the carbon content was low.^49^ The biochar produced by flash pyrolysis suffers low thermal stability and has minimal porosity.^48^

### 2.2 Hydrothermal carbonization

Hydrothermal carbonization (HTC) is an effective wet thermochemical process that converts biomass into biochar at moderate temperatures (150–400 °C) and high pressure (1.4–27.6 MPa) in an aqueous medium.^50^ In contrast to pyrolysis, hydrothermal carbonization (HTC) eliminates the need for biomass drying, rendering it energy-efficient and economically advantageous for the treatment of wet biomass.^51^ The process generates hydrochar at temperatures below 250 °C, which can be utilized for soil enhancement and carbon sequestration. At temperatures ranging from 250 to 400 °C, the HTC process gradually transitions toward hydrothermal liquefaction, resulting in the production of bio-oil for fuels and chemicals. At temperatures exceeding 400 °C, HTC results in the production of syngas for energy production.^52^ HTC-derived biochar possesses a highly porous structure, augmenting its adsorption capability for environmental applications such as waste-water treatment. The yield of hydrochar (27–66%) is depending upon the type of feedstock and processing conditions, exemplified by rice husk hydrochar (57.9% at 180 °C) compared to fruit waste hydrochar (27–38% at 190 °C). Fig. S2 depicts the morphological analysis of RH and RB, which shows well-defined pores and rough surface textures, contributing to its high surface area.^53,54^ Additionally, HTC-derived biochar performs as an optimal catalyst support due to its abundant oxygen-containing functional groups, enhanced porosity and capacity for metal adsorption. These properties facilitate the metal dispersion, inhibit agglomeration and amplify catalyst stability. The inclusion of metal ions (*e.g.*, Fe^2+^ and Fe^3+^) during HTC can alter the surface characteristics and porosity, hence improving its efficacy in hydrodeoxygenation (HDO) and hydrogenation processes. These attributes render HTC a sustainable and scalable method for biomass valorisation in green energy, catalysis and carbon-based products.^55^Table 1 shows the comparison of HTC and pyrolysis.

**Table 1:** Comparative overview of hydrothermal carbonization (HTC) and pyrolysis.^55^

Feature	Hydrothermal carbonization (HTC)	Pyrolysis
Temperature	150–350 °C	300–900 °C
Pressure	1.4–27.6 MPa	Atmospheric
Feedstock	Wet biomass	Dry biomass
Functional groups	Rich in –OH and –COOH	Fewer oxygen-containing groups
Catalyst support	High metal dispersion	Potential metal sintering

### 2.3 Gasification

Gasification is a high-temperature thermochemical process (>500 °C) that converts biomass into syngas, tars, and biochar in an oxygen-limited environment.^56,57^ The primary product, syngas, composed mainly of hydrogen (H_2_), carbon monoxide (CO), carbon dioxide (CO_2_), methane (CH_4_) and other hydrocarbons, serves as a versatile energy source for power generation, chemical synthesis, and fuel applications.^58^ Various reactor systems such as fluidized bed, entrained bed, moving bed, and fixed bed reactors are employed to optimize gasification efficiency and syngas quality. While syngas is the main output, biochar emerges as a valuable by-product with unique physicochemical properties.^59^ Unlike pyrolysis, which prioritizes biochar production, gasification yields a lower biochar output (10–15%).^60^ However, biochar formed through gasification exhibits greater stability, enhanced resistance to chemical oxidation and reduced particle size, making it highly suitable for carbon sequestration, soil enhancement, and pollutant adsorption.^61^ Additionally, gasification biochar typically has a higher ash content and a more porous structure, with well-defined channels observed through SEM analysis, leading to increased surface area and superior adsorption capacity. Elevated temperatures, particularly around 750 °C, further enhance porosity, resulting in larger pores and improved adsorption characteristics.^62^

### 2.4 Microwave-assisted pyrolysis

Microwave-assisted pyrolysis (MAP) is a sophisticated thermochemical method that improves the efficiency of biochar production while maintaining sustainability. In contrast to traditional pyrolysis, which depends on external heat transfer, MAP employs microwave radiation to internally heat biomass, leading to accelerated heating rates, enhanced temperature regulation and increased biochar yield.^63^ This distinctive heating method facilitates more uniform and selective heating, lowering thermal gradients and improving the reaction efficiency. Furthermore, MAP provides reduced energy usage, rendering it a cost-effective and ecological substitute for conventional pyrolysis methods. The key advantages of biochar include increased specific surface area and abundant pore structures, which provide more active adsorption sites and consequently improve its adsorption performance in environmental applications, water purification and soil improvement.^59^ MAP is acknowledged as an eco-friendly method owning to its diminished emission, decreased energy consumption and expedited processing duration, rendering it suitable for extensive biochar production.^64^ The quality and yield of biochar are contingent upon the type of feedstock, reaction condition and process parameters. For example, biosolid biochar at 300 °C achieved a yield of 90 wt%, while the yield of palm oil empty fruit bunch biochar using 600 W microwave power reached 94 wt%.^63^

### 2.5 Torrefaction

**Fig. 6:**
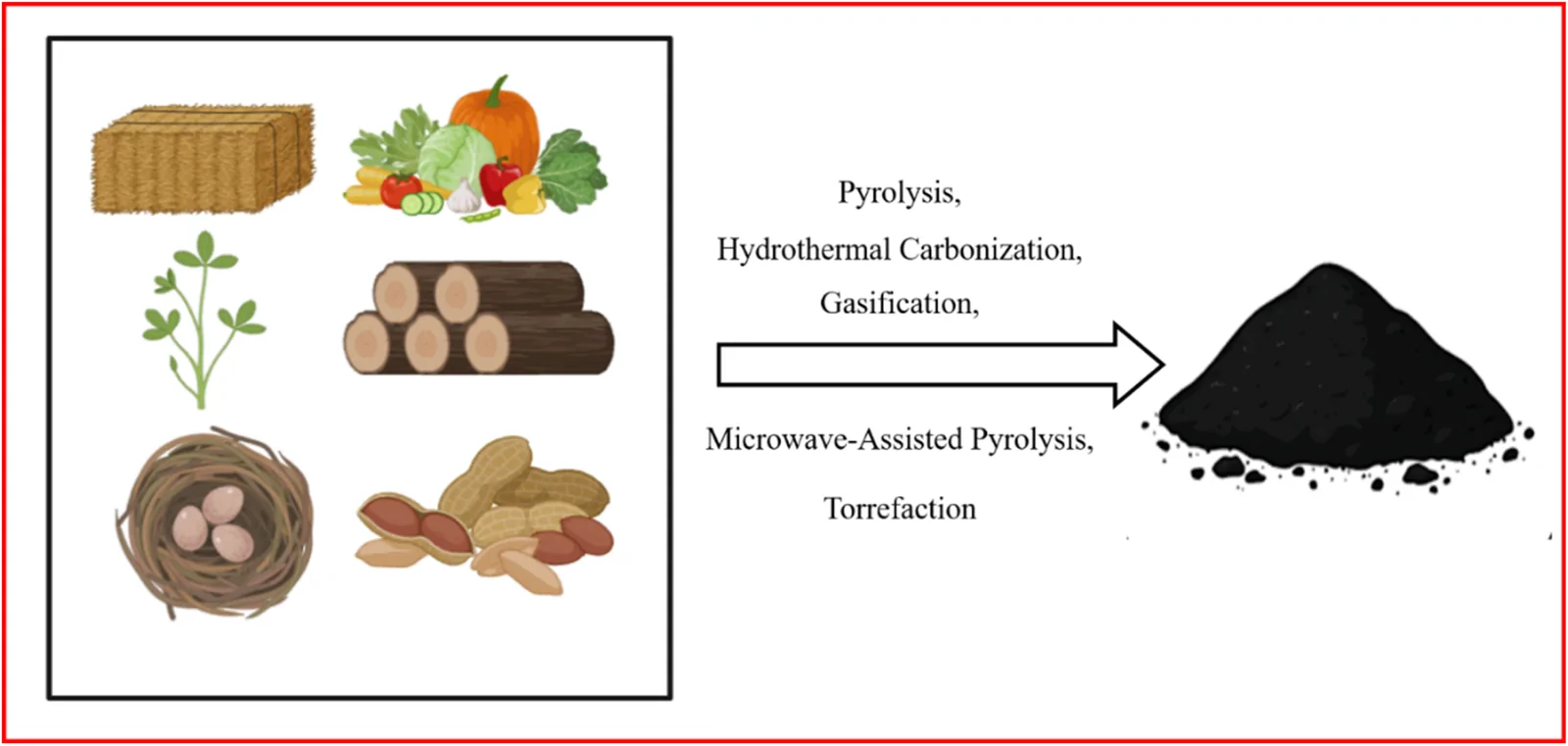
Methods for biochar preparation.

Torrefaction is a thermal conversion process carried out at moderate temperatures ranging between 200 and 300 °C under an inert or low oxygen environment. The process leads to the partial disintegration of the biomass, thus changing its characteristics in terms of physical and chemical properties.^64^ Torrefaction improves the energy content, low affinity to moisture, and the ability to grind the biomass, making it suitable for storage and transportation and conversion to fuel or feedstock. Although the main objective of torrefaction is to enhance the biomass for energy uses, the process results in the formation of biochar.^65^ The yield is usually between 48% and 80% depending on the process conditions, and the biochar contains less moisture and more calorific value than the biomass feed.^66^ The biochar preparation methods are presented in Fig. 6. An overview of various thermochemical processes for biomass conversion is presented in Table 2.

**Table 2:** Biochar preparation: optimization of methods and parameters

Process	Description	Temperature	Yield	Ref.
Slow pyrolysis	It is a thermal breakdown process in which the biomass is heated under an inert condition at a comparatively low temperature	400–750 °C	50–60%	37 and 38
Fast pyrolysis	Biochar can be generated through rapid heating	400°–650 °C	12%	44
Flash pyrolysis	The biochar production process is even more expedited than fast pyrolysis, with an exceedingly brief residence time	1000 °C	Below 10%	48
Hydrothermal carbonization	Converts biomass to biochar at elevated temperature and pressure in an aqueous medium	250–400 °C	50%	52 and 53
Gasification	Biomass conversion into syngas and biochar	>500 °C	10–15%	56 and 60
Microwave-assisted pyrolysis	Biomass is subjected to heating *via* microwave radiation in an inert atmosphere	300 °C	90–94%	67
Torrefaction	Thermal conversion process conducted at moderate temperatures under an inert atmosphere	200–300 °C	48–80%	65

## 3 Biochar–metal composite preparation and characterization methods

### 3.1 Preparation methods

#### 3.1.1 Chemical reduction

**Fig. 7:**
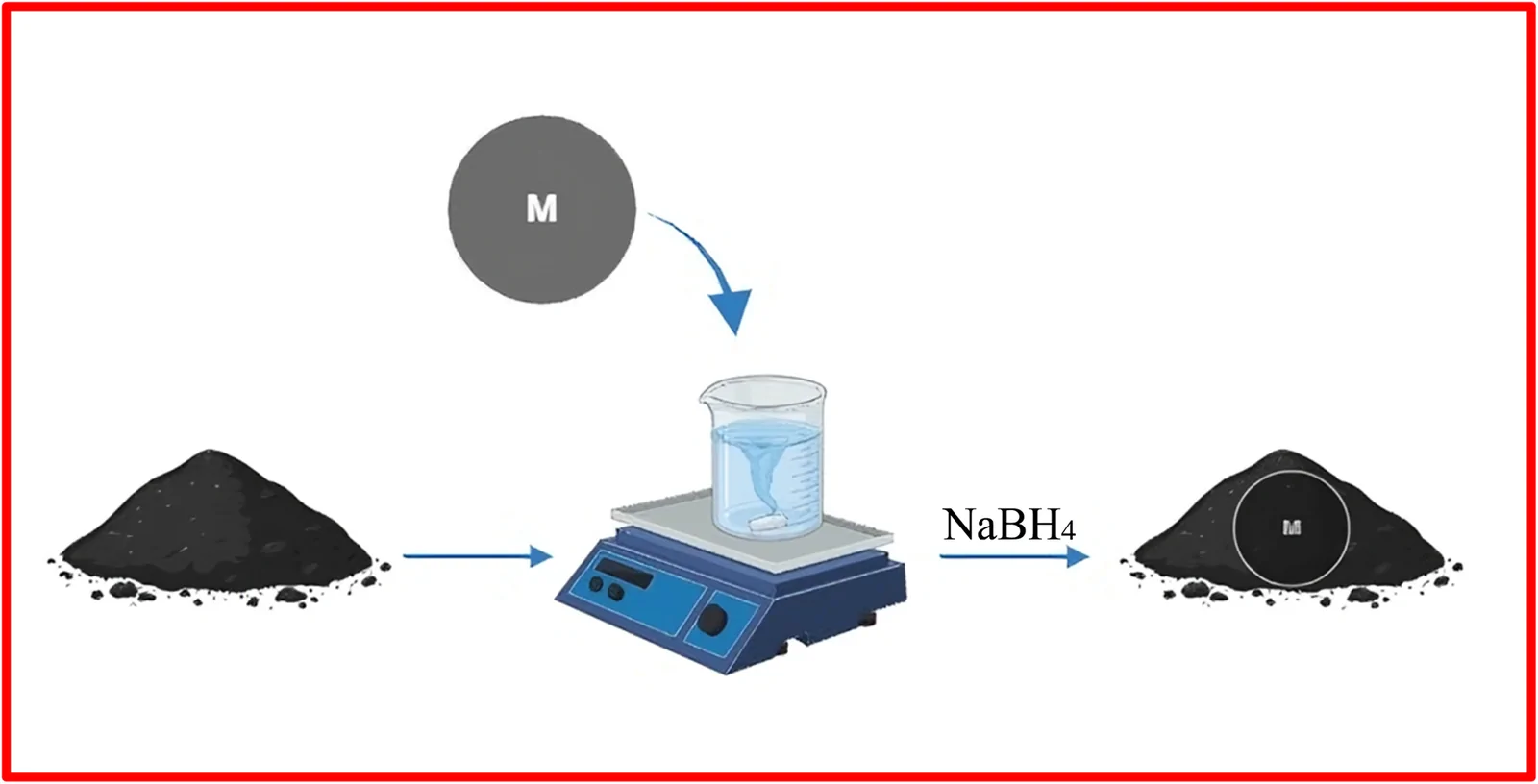
Schematic of the preparation of biochar–metal composites *via* a chemical reduction method.

Chemical reduction is a widely used method for preparing Biochar–metal composites because it enables rapid nanoparticle formation under mild conditions. In this method, metal precursors are reduced using external reducing agents such as sodium borohydride, hydrazine and sodium citrate. Typically, a metal salt solution is mixed with biochar, followed by the dropwise addition of the reducing agent.^68,69^ The reducing agent donates electrons to the metal ions, reducing them to their zero-valent metallic state. The resulting nanoparticles are deposited and uniformly anchored onto the biochar surface through interactions with its abundant oxygen-containing functional groups and porous structure.^70^ The physicochemical properties of biochar-supported metal composites prepared by chemical reduction are governed by metal concentration, metal incorporation, and the surface functional groups of the biochar. These factors significantly influence nanoparticle size, dispersion, surface area, and the availability of active sites.^71^ For example, biochar-supported nZVI composites have been reported to exhibit a surface area of 317.57 m^2^ g^−1^ compared with 26.7 m^2^ g^−1^ for pristine nZVI.^72^ Similarly, sodium borohydride reduction has been shown to produce a homogeneous distribution of nanoparticles in the nanometre range, whereas increasing Ni–Cu incorporation from 5% to 15 wt% increases the particle size from 2.3 ± 0.3 nm to 6.2 ± 1.2 nm. Excessive metal incorporation promotes nanoparticle growth and agglomeration, which can reduce metal dispersion and the availability of active sites.^73^ The preparation of metal–biochar composites *via* a chemical reduction method is illustrated in Fig. 7.

Compared with other methods, chemical reduction generally offers greater control over nanoparticle nucleation, dispersion, and metal incorporation, which is important for catalytic and adsorption applications. Its operational simplicity also makes it attractive for both laboratory-scale preparation and scale-up. However, it depends on reducing agents such as NaBH_4_, which increases the cost, generates chemical waste, and raises environmental concerns. These limitations constrain scalability and motivate the development of more sustainable reducing agents that minimise the environmental impact while maintaining high adsorption and catalytic activity.

#### 3.1.2 Phytoreduction (green reduction)

Phytoreduction, also known as green reduction, has emerged as a sustainable method for preparing biochar–metal composites using plant-derived phytochemicals as natural reducing agents. Bioactive compounds such as polyphenols, flavonoids, tannins, alkaloids, sugars, and organic acids reduce metal ions to metallic nanoparticles.^74^ This environmentally friendly approach eliminates the need for hazardous reducing agents, making it attractive for fabricating composites for environmental remediation. Typically, an aqueous plant extract is added to a metal precursor solution in the presence of biochar. During reduction, phytochemicals donate electrons to metal ions, forming nanoparticles immobilised on the biochar surface.^75^ Tran *et al.* prepared an Ag/biochar composite using *Citrus maxima* peel extract as the reducing agent. HRTEM analysis showed spherical AgNPs of 3–10 nm deposited on the biochar surface, and the composite exhibited high adsorption and antibacterial activity owing to the uniform distribution of Ag nanoparticles.^76^ Similarly, Hosny *et al.* prepared a phyto-fabricated Ag–Cu/biochar composite using *Atriplex halimus* biomass as both the biochar source and the reducing extract, with particle sizes of 25–45 nm this composite showed high catalytic activity for doxycycline removal compared with monometallic composites, attributed to synergistic electron transfer between Ag and Cu nanoparticles and improved structural stability during repeated use.^77^

Compared with chemical reduction, phytoreduction eliminates toxic reducing agents such as NaBH_4_ and hydrazine and offers lower chemical consumption and cost. However, achieving uniform nanoparticle dispersion and controlling particle size remain challenging, while optimising the precursor concentration, extract dosage, pH, and reaction time is also difficult. These factors can affect reproducibility, efficiency, long-term stability and scalability.

#### 3.1.3 Carbothermal reduction

Carbothermal reduction is a one-step approach in which biomass serves as both the carbon source and the reducing agent. During pyrolysis under an inert atmosphere, carbon from the biomass reduces metal precursors to metal nanoparticles while forming biochar, eliminating the need for external reducing agents such as NaBH_4_ or plant extracts and simplifying the preparation process.^78,79^ Typically, biomass is impregnated with metal salts prior to pyrolysis, and thermal decomposition drives reduction of the metal ions and formation of nanoparticles within the porous carbon structure.^80^ Huang *et al.* prepared several biochar-supported metal catalysts (Ni-, Fe-, Cu- and Ru-loaded) using one-step carbothermal reduction from lignin, soybean straw, and *Chlorella vulgaris*. The Ni-loaded lignin-derived biochar showed the highest catalytic performance, attributed to lignin's high fixed-carbon content, which promoted efficient carbothermal reduction and uniform nanoparticle dispersion (particle size = 32–51.2 nm). This route offers strong metal–support interactions, high stability, and elimination of external reducing agents, but typically requires high pyrolysis temperatures (>700 °C), resulting in high energy consumption, and catalyst properties remain strongly dependent on biomass composition and pyrolysis conditions.^81^

#### 3.1.4 Comparative analysis

The three preparation methods differ in preparation control, environmental sustainability, and scalability. Chemical reduction offers the greatest control over nanoparticle nucleation, size, and dispersion, making it suitable where precise particle size and metal loading are required, but at a higher chemical cost and environmental burden. Phytoreduction is the most sustainable route, replacing hazardous chemicals with plant-derived reducing agents, though variability in extract composition and slower reaction kinetics can limit reproducibility and scale-up. Carbothermal reduction is a solvent-free, one-step route that simplifies preparation and promotes strong metal–support interaction but requires greater energy input. The optimal route therefore depends on the intended application. Chemical reduction is suitable for applications demanding precise size control, phytoreduction is preferred when green synthesis is the priority, and carbothermal reduction is attractive for robust, one-step fabrication.

### 3.2 Characterization of biochar–metal composites

**Fig. 8:**
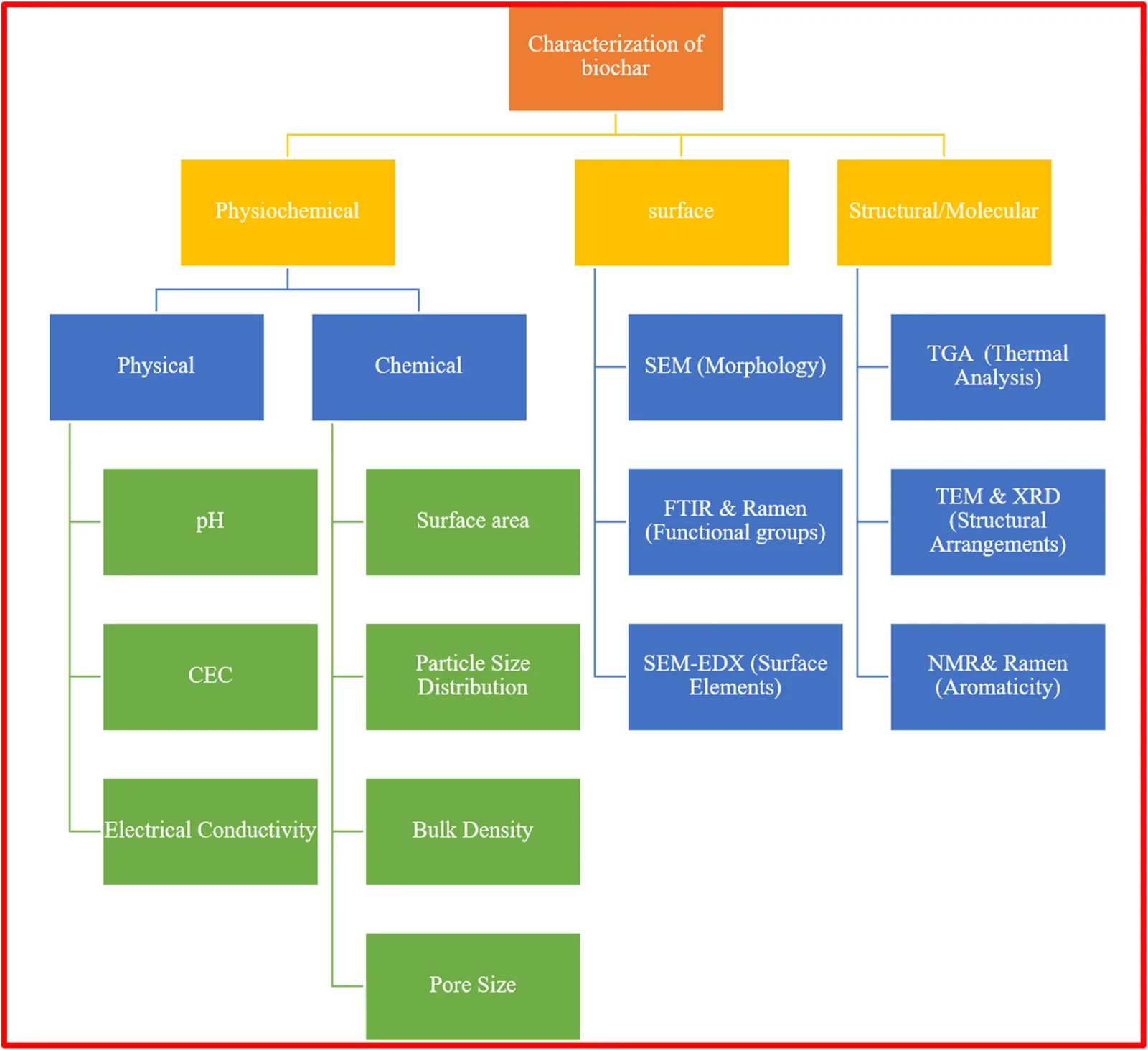
Overview of the major characterization techniques used in analyzing biochar–metal composites.

Biochar–metal composites can be characterized using a variety of techniques that reveal their optical properties, surface functionality, electronic structure, and other essential physicochemical attributes. Fig. 8 presents an overview of the major characterization techniques used for analyzing biochar composites.

#### 3.2.1 UV-visible (UV-vis) spectroscopy

UV-vis spectroscopy is one of the prominent methods to examine the optical characteristics, surface functionality and electronic structure of materials.^82^ It offers a crucial understanding of the dimensions and functional groups on biochar surfaces, which are vital for assessing their reactivity. This method is especially effective in verifying the successful conversion of metal salts into nanoparticles and monitoring catalytic reactions to assess the efficacy of biochar–metal composites. The UV-vis Diffuse Reflectance Spectroscopy (UV-DRS) technique was employed to investigate the optical characteristic of biochar–metal composites. UV-DRS investigation revealed that TiO_2_-modified biochar composites displayed significant UV-adsorption, with Ti-KBC exhibiting the lowest bandgap energy of 2.41 eV. An augmented bandgap increases visible light adsorption, resulting in enhanced photocatalytic efficiency.^83^ Likewise, UV-DRS analysis of Ag@biochar validated the synthesis of silver nanoparticles, evidenced by the detection of a surface plasmon resonance (SPR) peak at 420 nm, as shown in Fig. S3. The deposition of AgNPs on biochar decreased to 1.9 eV relative to pure biochar, hence improving visible light absorption.^84^ The optical properties revealed by UV-vis spectroscopy correlate directly with catalytic performance: a reduced bandgap facilitates electron excitation under visible-light irradiation, decreasing electron–hole recombination and increasing charge-transfer efficiency during photocatalytic reactions, while the surface plasmon resonance of metal nanoparticles enhances light absorption and generates energetic electrons that further improve the photocatalytic degradation efficiency and reaction kinetics.

#### 3.2.2 Fourier transform infrared (FT-IR) spectroscopy

FT-IR spectroscopy is a key tool for determining the functional groups on the surface of biochar, and these surface groups are essential for the interaction of biochar with diverse molecules.^85^ The functional groups in biochar differ based on the pyrolysis temperature, as biochar generated during pyrolysis often comprises a higher amount of volatile matter.^86^ For example, the FT-IR study of the biochar–iron composites (PBC, RBC, Fe-PBC and Fe-RBC) yields essential information regarding their functional group and adsorption mechanisms. As shown in Fig. S4, the composites displayed significant transitions of functional groups, including OH, C=O, C–O–C and Fe–O stretching vibrations, which were crucial for their efficacy. Significantly, Fe-PBC exhibited a peak at 562 cm^−1^ (Fe–O stretching) affirming the effective incorporation of iron. However, there is a shift in peaks (OH, C=O and Fe–O), confirming the participation of these groups in the adsorption process. It is facilitated by mechanisms such as electrostatic attraction, hydrogen bonding and π–π interaction.^87^ The functional groups identified by FT-IR spectroscopy also influence the catalytic performance by mediating interactions between the metal nanoparticles, the biochar support, and reactant molecules. Hydroxyl, carboxyl and carbonyl groups facilitate reactant adsorption and metal–support interaction and increase the electron-transfer capacity of the catalyst. Overall, an abundance of surface functional groups improves adsorption capacity, catalytic activity, and catalyst stability.

#### 3.2.3 Brunauer–Emmett–Teller (BET) analysis

BET analysis is an essential method for assessing the surface area and the porosity of materials such as biochar, offering critical insights into their adsorption potential, as larger surface areas and higher porosity generally enhance biochar–metal composite performance in adsorption processes.^88,89^ The BET study of biochar–metal composites demonstrates the impact of structural alterations on their characteristics. Fig. S5 shows the BET surface area of raw CEB (*Commelina erecta* biomass), recorded at 772.96 cm^2^ g^−1^, which markedly diminished to 163.46 cm^2^ g^−1^ for the CEB-AgNP nanocomposite, signifying a decrease in porosity attributable to the addition of silver nanoparticles. The pore volume decreased from 0.0175 cm^3^ h^−1^ (CEB) to 0.00072 cm^3^ g^−1^ (CEB-AgNP), indicating a reduction in accessible pore space. Notably, upon calcination at 673 K, the BET surface area of the CEB-AgNP nanocomposite elevated to 331.57 cm^2^ g^−1^, while the pore volume grew to 0.0039 cm^3^ g^−1^.^90^ These results demonstrate that the surface area and pore structure jointly govern adsorption and catalytic performance. A high specific surface area and well-developed pore structure provide abundant adsorption sites and facilitate reactant diffusion to active metal sites and although metal nanoparticle deposition can partially reduce the surface area by occupying the pore structure, the resulting active sites enhance electron transfer and catalytic efficiency. An optimal balance between porosity, surface area, and active-site availability is therefore essential for efficient adsorption and catalytic performance.

#### 3.2.4 Scanning electron microscopy (SEM)

**Fig. 9:**
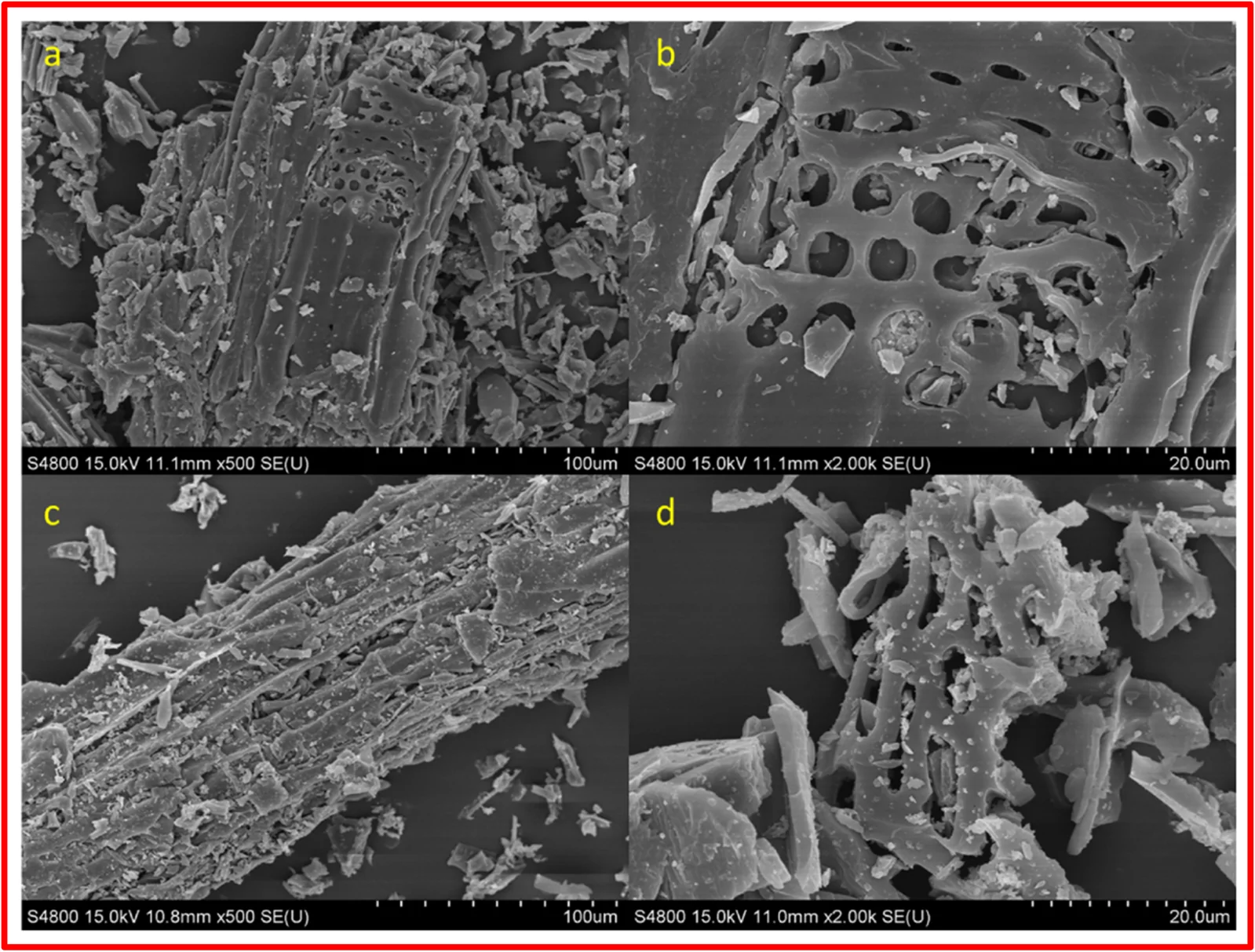
SEM topographical images of AgW (a and b) and AgH (c and d).^93^

SEM is a crucial instrument for examining the surface morphology of biochar–metal composites, providing intricate visualization of pore distribution and surface texture.^91,92^ The SEM study of biochar-supported silver composites demonstrated significant variation in shape and nanoparticle distribution between those produced using carbothermal reduction (AgH) and the wet chemistry approach (AgW), as shown in Fig. 9. AgH composites displayed rougher surfaces characterized by twisted carbon sheets and distorted pores, resulting from elevated preparation temperatures and a decreasing hydrogen gas environment, whereas AgW composites exhibited smoother surfaces. Moreover, the silver nanoparticles in AgH exhibited a more uniform distribution and were incorporated more deeply inside the biochar matrix, whereas in AgW they predominantly resided on the surface. The enhanced integration in AgH resulted in structural alteration to the charcoal matrix, improving electron transfer, charge carrier separation and catalytic activity.^93^ When scanning electron microscopy (SEM) combined with energy-dispersive X-ray (SEM-EDX) spectroscopy, it serves as an effective tool for analyzing the elemental composition of biochar–metal composites, elucidating the presence of adsorbed pollutants, especially metals.^94^ The EDX examination of the bentonite/silver nanoparticle-alginate (Bent/AgNPs-Alg) bio-nanocomposites, as shown in Fig. S6(a), revealed their elemental composition comprising oxygen (54.02 wt%), carbon (18.9 wt%), aluminium (6.07 wt%), silicon (20.47 wt%) and silver (0.54 wt%) prior to adsorption. As shown in Fig. S6(b), the adsorption of methylene blue (MB) dye grew notably to 36.05 wt%, thereby validating the efficient adsorption of the dye onto the composite surface. The silver nanoparticles showed stability within the composite matrix, with their content exhibiting negligible variation (0.54 wt% prior to adsorption and 0.49 wt% subsequent to adsorption).^95^ These SEM and SEM-EDX results illustrate the structure–performance relationship in biochar–metal composites. The uniformly dispersed metal nanoparticles create a large number of accessible catalytic sites and facilitate efficient electron transfer, supporting high catalytic activity, while a porous surface morphology improves the availability of adsorption sites, and stable, uniformly distributed nanoparticles after adsorption (as confirmed by EDX) indicate resistance to leaching and suitability for repeated use.

#### 3.2.5 X-ray diffraction (XRD)

**Fig. 10:**
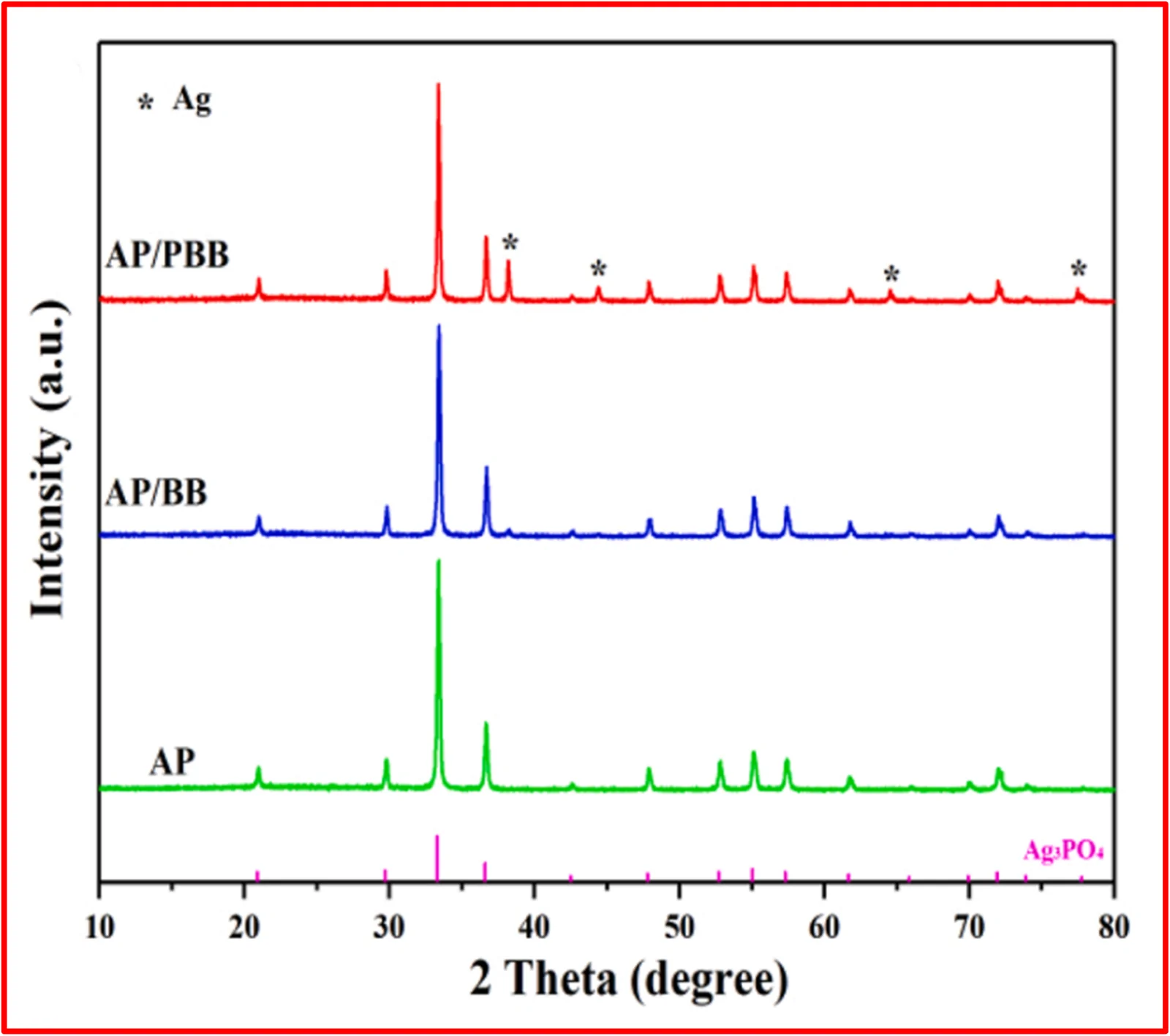
XRD patterns of AP, AP/BB and AP/PBB samples.^97^

X-ray diffraction (XRD) is a vital approach for examining the crystallinity and structure of biochar–metal composites, offering significant insights into their metal composition and crystalline phases.^96^ XRD examination of the AP/PBB composite identifies cubic Ag_3_PO_4_ as the predominant phase, and Fig. 10 reveals metallic silver (Ag^0^) peaks at 38.2°, 44.4°, 64.5° and 77.5°, signifying the *in situ* reduction of Ag ions by biochar. The increased intensity of Ag peaks in AP/PBB relative to AP/BB underscores the contribution of porous bamboo biochar (PBB) in facilitating superior dispersion of Ag_3_PO_4_ nanoparticles and enhanced interfacial contacts.^97^ These XRD results indicate that the crystallinity and phase composition of biochar–metal composites are directly linked to their adsorption and catalytic performance. The well-defined metallic phases provide abundant active catalytic sites and contribute to enhanced catalytic activity, improved stability, and sustained performance over repeated cycles.

#### 3.2.6 Thermogravimetric analysis (TGA)

Thermogravimetric analysis (TGA) is an essential thermal analytic method employed to evaluate the physicochemical characteristic of biochar–metal composites in relation to temperature. It offers insights into their thermal stability, breakdown characteristics and the composition.^98^ The TGA analysis of the biochar/Fe_3_O_4_@SiO_2_-Ag nanocomposite identified three discrete stages of weight reduction: approximately 4.59% below 150 °C due to the evaporation of adsorbed water, around 10% between 200 and 500 °C resulting from the dehydration of hydroxyl groups and the decomposition of organic matter and approximately 20.75% above 500 °C linked to thermal phase transformations, as shown in Fig. S7. The composite maintained approximately 65% of its residual mass at 950 °C, demonstrating exceptional thermal stability. This strong thermal performance highlights its appropriateness for catalytic applications in a high-temperature environment.^99^ These results indicate that the thermal stability of biochar–metal composites contributes directly to their catalytic durability. The high thermal stability allows the catalyst to retain its structural integrity and active sites under reaction conditions, minimising deactivation during repeated use and supporting improved reusability and operational reliability.

## 4 Mechanisms of 4-nitrophenol adsorption and catalytic reduction on biochar–metal composites

Biochar–metal composites have emerged as highly efficient materials for the removal of organic pollutants from contaminated water, owing to the high surface area and abundant functional groups of biochar combined with the catalytic activity of metal nanoparticles. Their removal of contaminants involves both physicochemical and catalytic processes. Among these, pollutants are adsorbed onto the composite surface through interactions such as π–π interaction, electrostatic attraction, hydrogen bonding, pore filling, and van der Waals forces. In addition, the incorporated metal nanoparticles provide catalytic active sites that facilitate the reduction of contaminants, particularly 4-nitrophenol (4-NP), in the presence of sodium borohydride (NaBH_4_). The following subsections discuss the adsorption and catalytic behaviour of biochar–metal composites and their removal mechanisms for 4-nitrophenol.

### 4.1 Adsorption

**Fig. 11:**
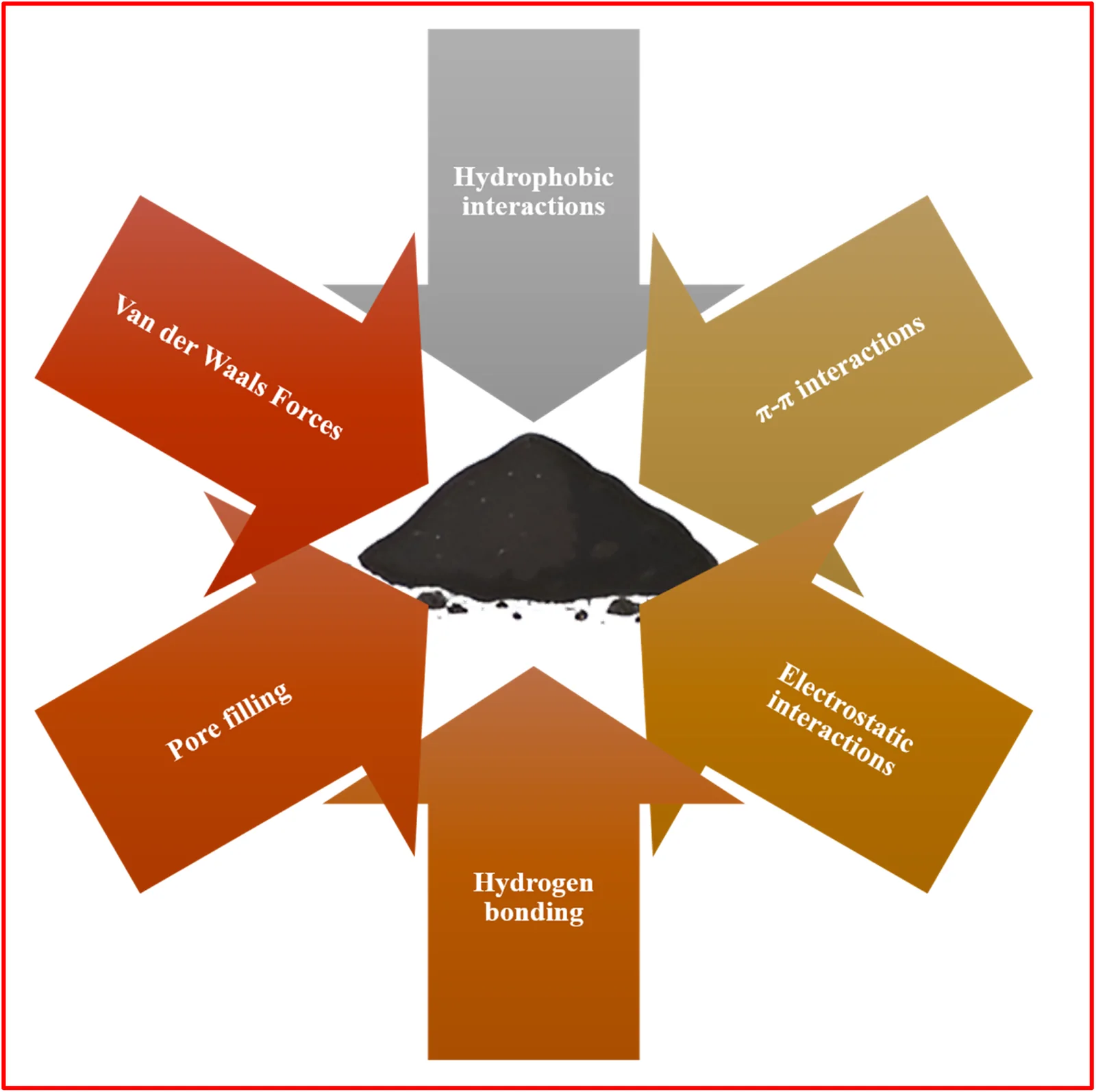
Key interaction mechanisms involved in the adsorption process on biochars.

Adsorption occurs between two phases, such as liquid–solid or gas–solid. The adsorbent is the material that provides the surface, while the adsorbate is the substance that attaches to the surface of the adsorbent.^100^ Adsorption is generally categorized into two types *viz.*, physisorption and chemisorption. On the one hand, chemisorption involves a chemical interaction between the adsorbate and the adsorbent, often resulting in the formation of new chemical bonds.^101^ On the other hand, physisorption involves physical interactions such as van der Waals forces (weak intermolecular forces) between the adsorbate and the adsorbent.^102^ Adsorption, in the decontamination of organic contaminants in aqueous solutions, is a promising alternative if the sorbent is inexpensive and does not need pre-treatment. In environmental sustainable remediation, adsorption technologies are extensively employed to eliminate toxic chemical pollutants from streams, particularly those that are effectively not impacted by traditional biological wastewater treatment.^103^ Apart from that, the modification of biochar as an inexpensive adsorbent is essential to maximize its surface utilities and holds immense potential to be used in water purification. Adsorbent-to-adsorbate ratio, adsorbent–adsorbent interaction, adsorbent surface area, temperature, pH, and contact time are some of the parameters that impact the adsorption efficiency.^104,105^Fig. 11 shows the major interaction mechanisms between biochar and adsorbates, including hydrophobic interactions, π–π interactions, electrostatic interactions, hydrogen bonding, pore filling, and van der Waals forces.

**Fig. 12:**
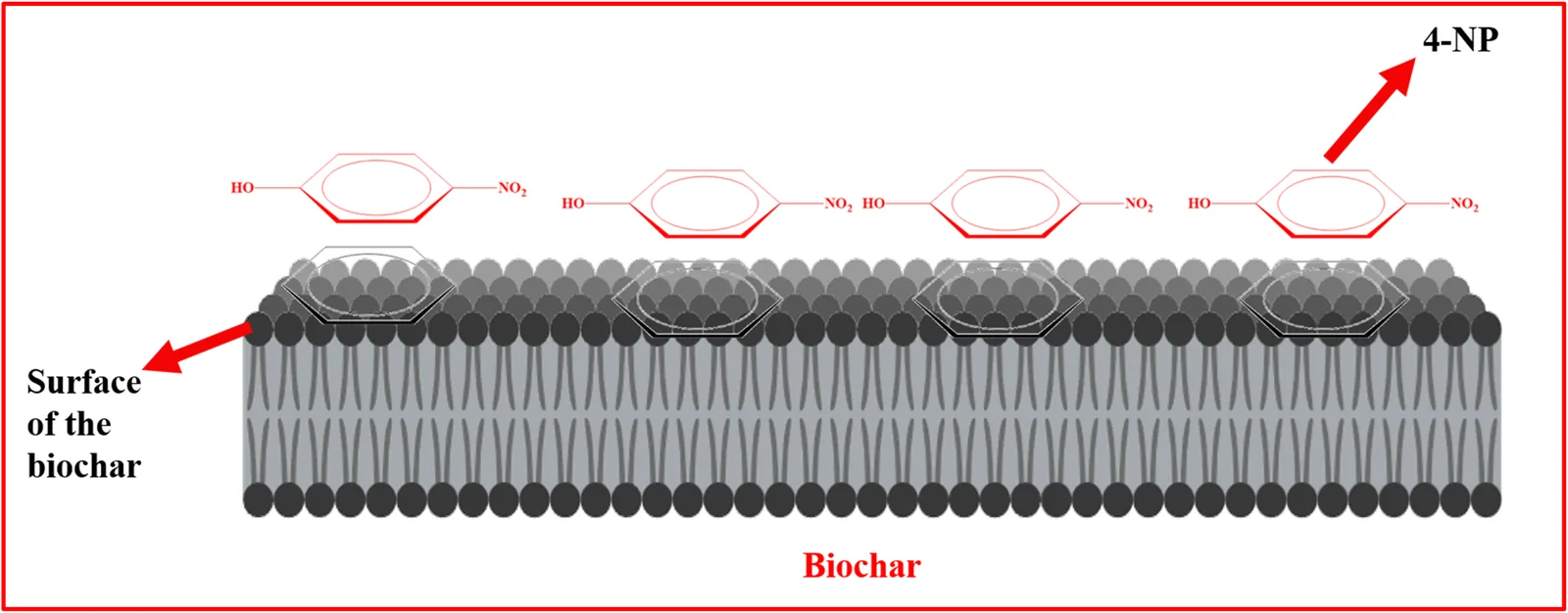
Schematic of the surface interaction between 4-nitrophenol (4-NP) molecules and biochars.

The removal of 4-nitrophenol (4-NP), an organic pollutant, from water by using biochar–metal composites is a major liquid–solid adsorption process.^106^ The adsorption between the surface of the biochar–metal composite and 4-NP involves different interactions such as hydrophobic interaction and π–π stacking.^107^ The hydrophobic interaction between the biochar–metal composite surface and 4-NP depends on the non-polar nature of the composite surface and the molecule of 4-NP. The hydrophobic surfaces of the aromatic carbon-bearing biochar–metal composite adsorb the non-polar moiety of 4-NP, thereby decreasing the free energy of the system and the process of adsorption.^108^ The aromatic rings available in both the biochar–metal composite and 4-NP also provide room for π–π stacking interaction, a non-covalent bond, further contributing towards effective 4-NP adsorption. This occurs when the delocalized π-electrons in the aromatic rings overlap, leading to a stable attraction. This interaction is particularly important for adsorbing aromatic compounds like 4-NP.^109,110^ The surface interaction between 4-nitrophenol (4-NP) molecules and biochar is illustrated in Fig. 12.

**Fig. 13:**
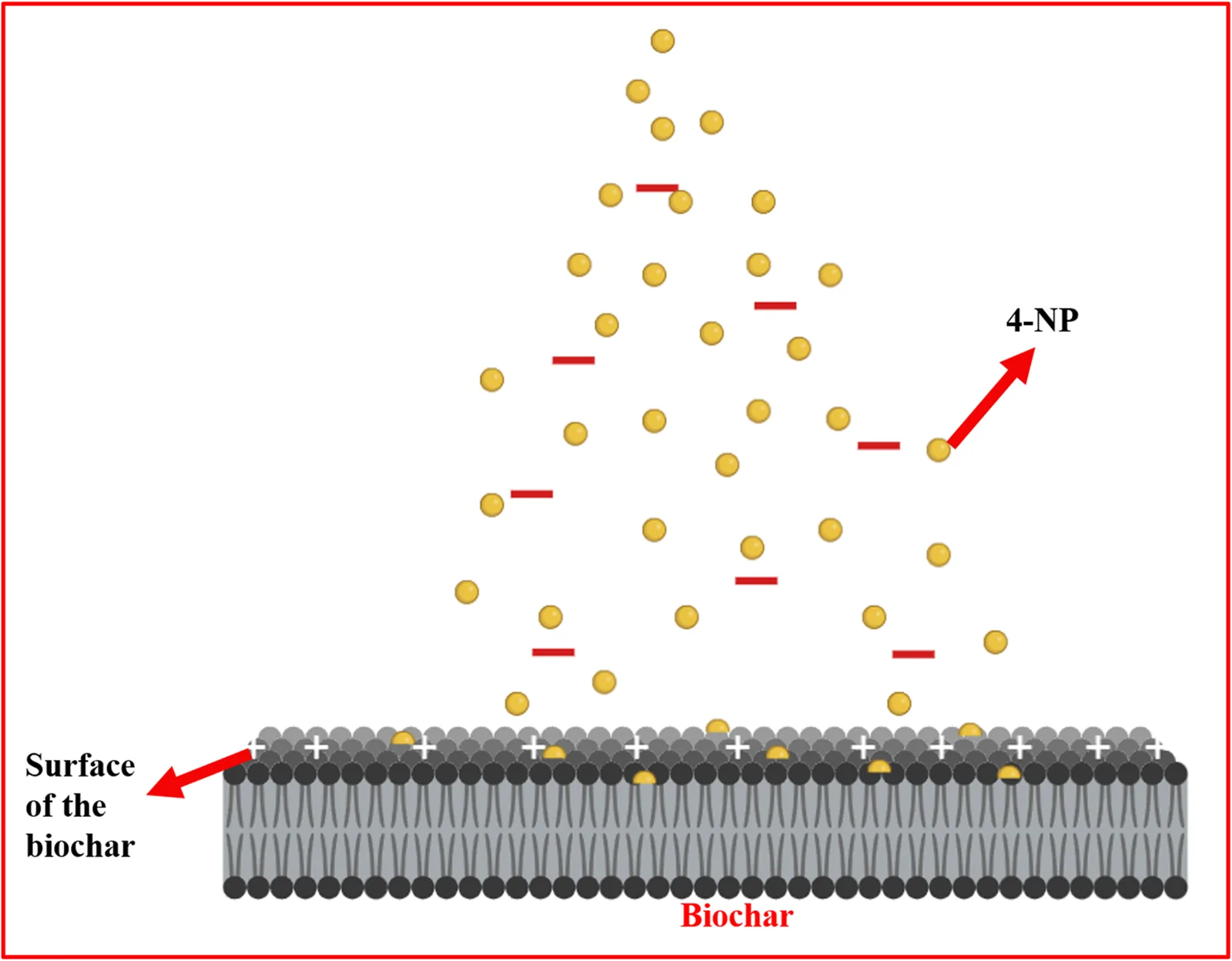
Illustration of the electrostatic interaction between 4-nitrophenol (4-NP) molecules and the surface of biochars.

The electrostatic interaction of biochar–metal hybrids with 4-nitrophenol (4-NP) is extremely pH-sensitive to solution pH, affecting the ionization state of 4-NP and the surface charge on the composite. At low pH, 4-NP largely occurs in the neutral molecular form. At higher pH, it is ionized and occurs in the nitrophenolate anion form. When the net charge on the composite surface of the biochar–metal is positive, most commonly by the protonation of the functional groups, it has the ability to electrostatically attract these negatively charged nitrophenolate ions and improve adsorption.^109^ Electrostatic interaction between 4-nitrophenol (4-NP) molecules and the surface of biochar is shown in Fig. 13.

**Fig. 14:**
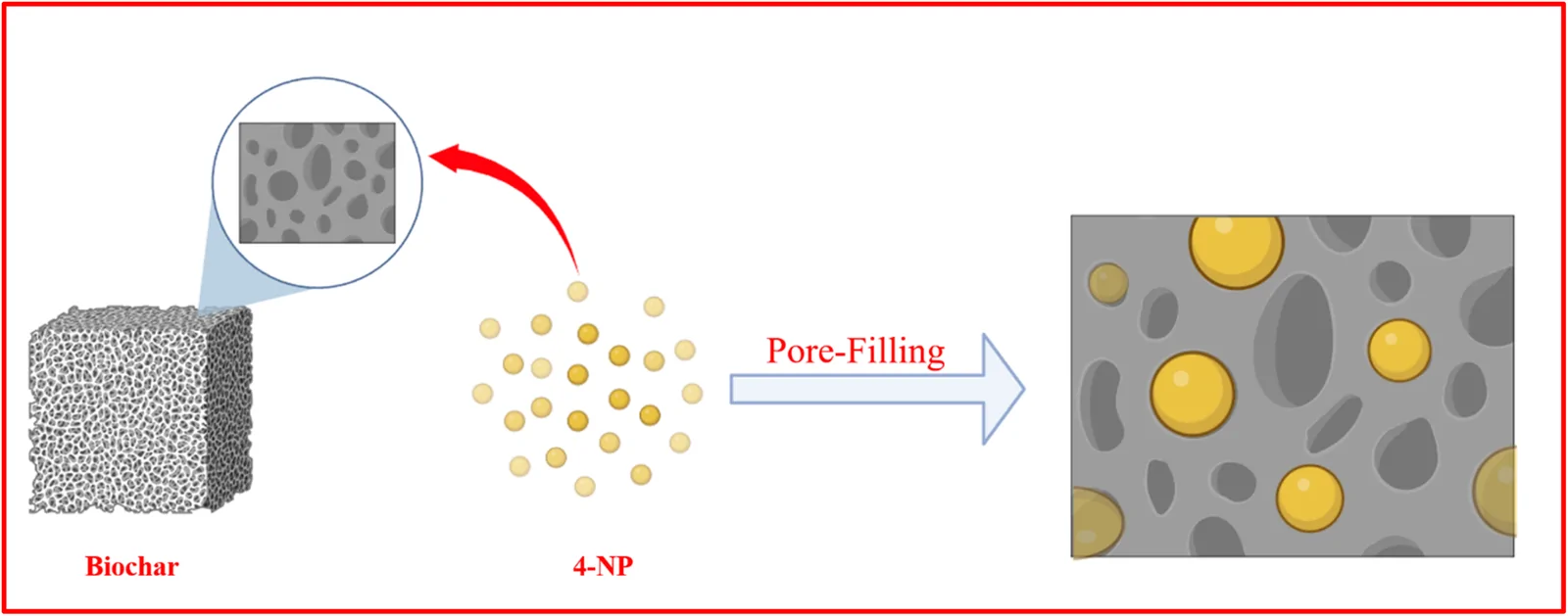
Schematic of the pore-filling mechanism of 4-nitrophenol (4-NP) into the porous structure of biochars.

**Fig. 15:**
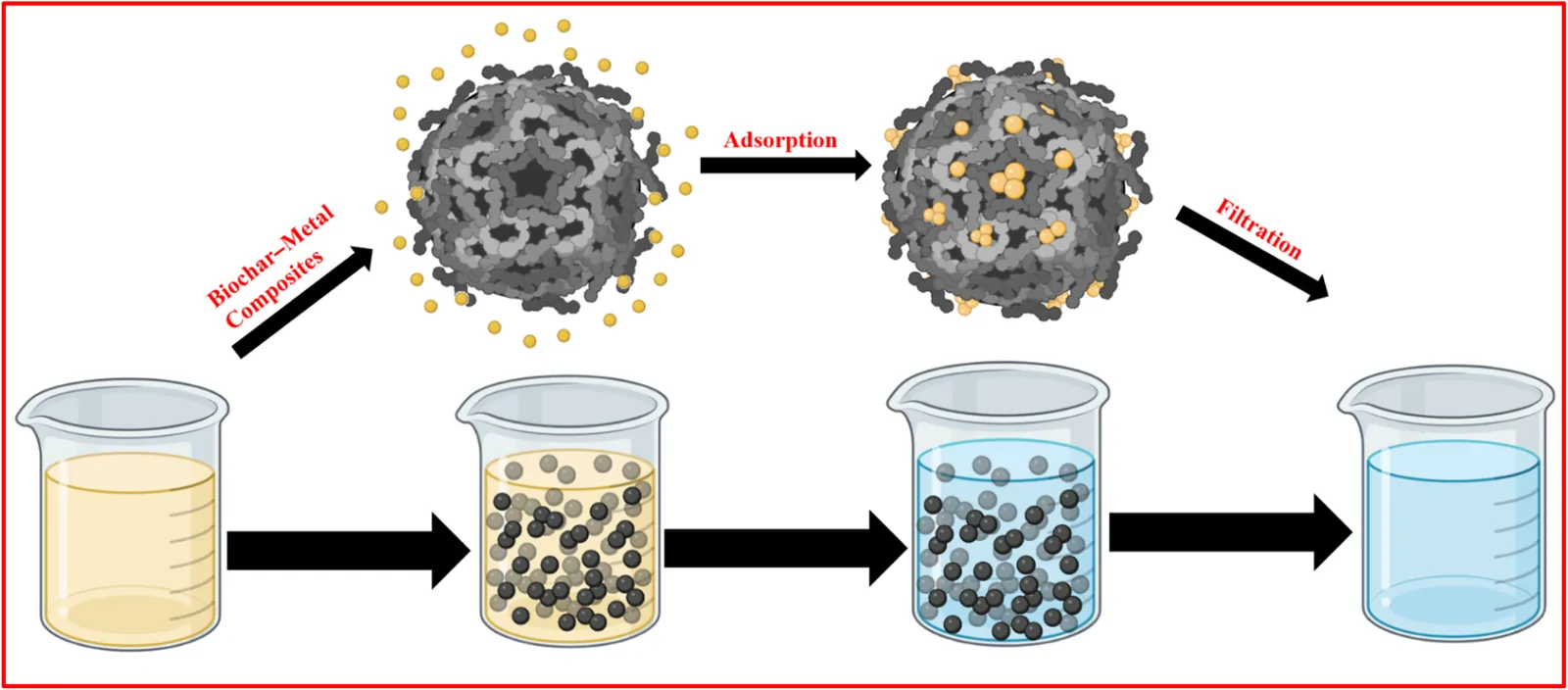
Schematic of the adsorption mechanism of 4-nitrophenol (4-NP) onto biochar–metal composites.

Hydrogen bonding is also responsible as a factor for the process of adsorption. This happens between oxygen-functional groups present in the biochar–metal composite (hydroxyl and carboxyl functional groups) and hydroxyl or nitro functional group present in 4-NP. The interaction plays an especially important role in polar functional group-rich biochars and helps to stabilize adsorbed 4-NP molecules.^111^ The porosity of biochar also enhances 4-NP adsorption. Because of their small molecular size, 4-NP molecules can easily penetrate the micro- and meso-pores of biochar, usually with diameters less than 2 nm to 50 nm. The pore-filling promotes physical adsorption on inner surfaces and greatly improves the overall adsorption capacity, especially in highly porous biochars.^112^Fig. 14 depicts the pore-filling mechanism of 4-nitrophenol (4-NP) into the porous structure of biochar. While less strong than other forces, van der Waals forces also stabilize 4-NP on the surface of biochar. The weak non-specific forces are due to transient induced dipoles between molecules and add to the overall process of adsorption.^113,114^ The schematic of the adsorption of 4-nitrophenol (4-NP) onto biochar–metal composites is shown in Fig. 15.

Five pine sawdust biochars (PC300, PC400, PC500, PC600, and PC700) were prepared under different pyrolysis temperatures ranging from 300 to 700 °C. PC600 and PC700 have adsorption capacities of 111.713 mg g^−1^ and 117.165 mg g^−1^, respectively. Low-temperature-prepared samples' adsorption is usually because of electrostatic interaction, but the prevailing adsorbate in hydrogen bonding and π–π interaction is present in the form of samples prepared at high temperatures, on which the temperature and pH can have impact.^109^ BC-PFP biochar indicated that 4-NP adsorption is attributed to the spontaneous and exothermic process with an adsorption capacity of 2.5 g L^−1^ and a contact time of 60 minutes. biochar–metal composites have higher adsorption capacity compared to the virgin biochar.^115^ Iron- and zinc-doped biochars were initially prepared by Wang *et al.* using thermal pyrolysis of wood residues under a nitrogen (N_2_) environment. The prepared adsorbent was studied for efficacy in 4-NP removal from aqueous solutions. The results showed that the maximum adsorption capacity of Fe/Zn-doped biochar for PNP was 170 mg g^−1^. The adsorption performance was influenced by several factors such as solution pH, ionic strength, and temperature. The adsorption of PNP onto the adsorbent was mainly attributed to the hydrophobic interaction.^116^ Temperature, hydrophobic contact, ionic strength, and pH were the major factors controlling the spontaneous endothermic process of 4-NP adsorption. The desorption efficiency of 4-NP by peanut shell- and green tea extract-derived iron/copper biochar (Gt-BC@nZVI/Cu) was 93% after 30 min but reduced to 47.32% after three cycles. 4-NP adsorption was dependent on co-existing ions, humic acid, temperature, and pH.^117^ The Cu/Ni composite materials of biochar obtained from olive husk were 156 mg g^−1^ and 143 mg g^−1^ for Ni–AC and Cu–AC, respectively. van der Waals forces and hydrogen bonds are the forces acting during adsorption.^111^

Overall, these findings indicate that a large surface area alone does not guarantee high adsorption activity; rather, the nature of the supported metal, the distribution of surface functional groups, pore structure, and the dominant adsorption mechanism together determine the adsorption efficiency across these systems. The GT-BC@nZVI/Cu system, for example, operates effectively at neutral pH; an operational advantage for practical wastewater treatment, yet its comparatively low adsorption capacity (35.21 mg g^−1^) suggests that its available adsorption sites or interaction mechanisms are less effective than those of the other systems compared here. Because its BET surface area and pore volume have not been reported in the original study, a direct structural comparison with the other adsorbents is not possible. Table 3 summarises the biochar–metal composites discussed and their adsorption capacities. The comparative adsorption capacities of biochar–metal composites for 4-nitrophenol removal are illustrated in Fig. S8.

**Table 3:** Biochar composites and their adsorption properties

Biochar–metal composites	Surface area (m^2^ g^−1^)	Pore volume (cm^3^ g^−1^)	Adsorbent dosage (g)	pH	Contact time (h)	Adsorption capacity (mg g^−1^)	Reusability	Ref.
PC 600	432.734	0.233	0.1	3–11	48	111.713	NR	109
PC 700	398.864	0.227				117.165		
Fe/Zn biochar	518.540	0.069	0.015	3	37	170.00	NR	108
GT-BC@nZVI/Cu	NR	NR	0.01	7	0.5	35.21	3	117
Ni–Ac	770.480	0.368	0.1	4	1	156.00	NR	111
Cu–Ac	625.841	0.308				143.00		
Ca-MPBC	5.460	NR	0.05	7	4	375.18	NR	118
MBC_2_@ZT_BM_	NR	NR	0.05	2	0.75	196.140	3	119
HNBC-3	1500.200	1.410	0.02	7	24	365.60	4	120
BSW	38.000	NR	1	6	1.5	12.00	NR	121
ACBC	91.000	1.700	0.5	5	0.08	79.36	6	122
MFBC	14.000	7.100				106.38		

### 4.2 Catalytic reduction of 4-nitrophenol (4-NP)

**Fig. 16:**
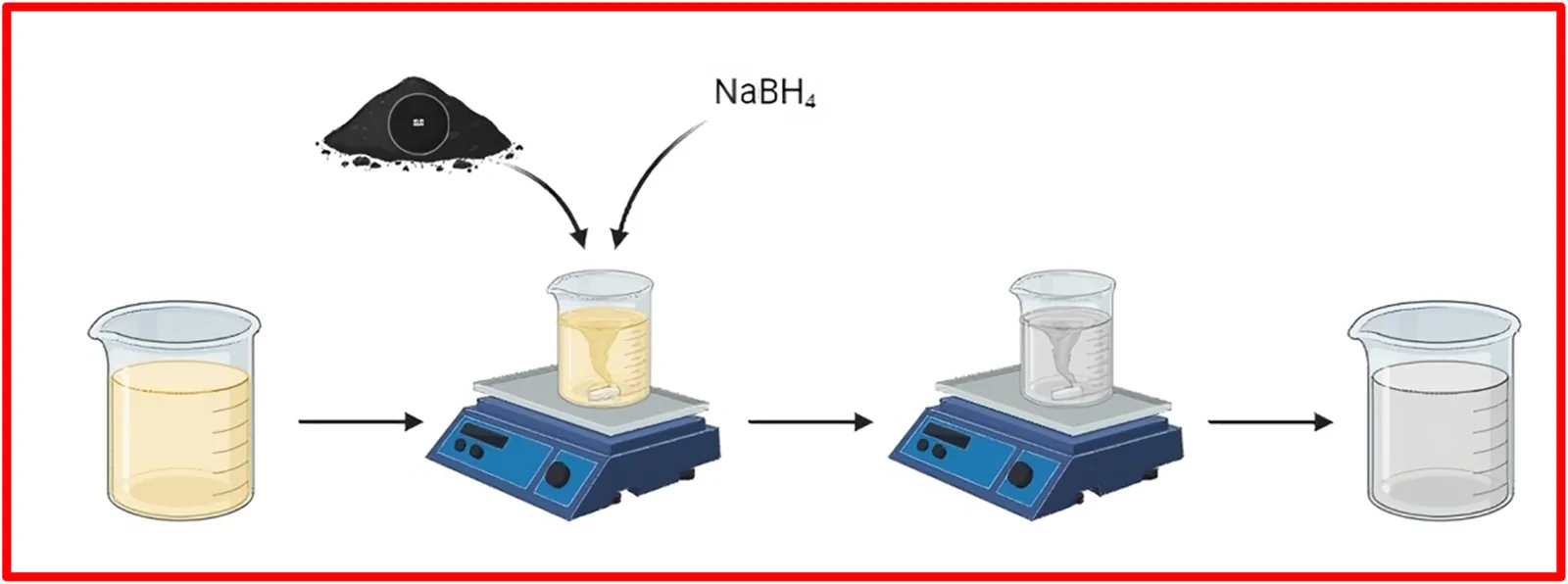
Catalytic reduction of 4-nitrophenol by biochar–metal composites.

The catalytic reduction of 4-nitrophenol (4-NP) has been chosen as a classical reaction to explore the catalytic propensity of various metal composites including silver nanoparticles, because it is straightforward and can be easily monitored by UV-vis spectroscopy under ambient conditions. The reduction of 4-NP can be easily identified even with the naked eye because it undergoes an apparent color change, *i.e.*, from yellow to colourless.^123,124^ The catalytic reduction of 4-NP using biochar–metal composites is shown in Fig. 16.

Ramirez *et al.* prepared a series of novel Cu, Co, Mn, and Ni BC-supported catalysts. The performance of these catalysts was evaluated for the catalytic reduction of 4-nitrophenol (4-NP) to 4-aminophenol (4-AP) in an aqueous solution in the presence of NaBH_4_ as a reducing agent. The Mn and Ni catalysts were not active under the studied reaction conditions. The 4-NP : NaBH_4_ mass ratio of the catalyst was also optimized for the Cu-based catalysts BC1CuOx and BC2CuOx, and the reduction of 4-NP was complete within 2.1 and 0.80 minutes, respectively. BC1CuOx showed the highest rate constant (*k*_n_) due to higher metal dispersion on the support, while BC2CuOx showed the highest apparent rate constant (*k*_app_) due to a higher metal content. BC_2_CuOx leaching test and reuse in five cycles of reaction showed that the catalyst is stable and reusable without any loss of activity. The findings indicate that Cu oxide-BC-supported catalysts are suitable for 4-NP degradation into 4-AP from wastewater.^125^

Baye *et al.* synthesized FeCx-coated carbon sheets (FC) *via* pyrolysis of waste biomass and used the sheets as a bifunctional catalyst for the reduction of 4-nitrophenol (4-NP). The findings indicated that FC900, synthesized at a calcination temperature of 900 °C, exhibited higher electrocatalytic and catalytic activity toward the detection and reduction of 4-NP. This improved performance was due to weaker FC900 4-NP interactions, allowing for easier desorption and adsorption of the reactants, quicker diffusion of 4-NP on the FC900 surface, and more efficient charge transfer kinetics. FC900 also demonstrated improved catalytic activity towards reducing 4-NP, in which it reduced the substrate totally within 8 minutes and had a rate constant of 0.42 min^−1^. Additionally, FC900 showed moderate stability during the catalytic reduction process.^126^

Cho *et al.* prepared a new nitrogen-doped copper–biochar (N–Cu–biochar) material through pyrolysis of glucose in the presence of Cu(ii) and melamine. N–Cu–biochar served as an efficient catalyst in the reduction of 4-NP by NaBH_4_. The catalytic activity of N–Cu–biochar was investigated under different conditions of NaBH_4_ concentration, dosage of biochar, and 4-NP concentration. N–Cu–biochar exhibited greater catalytic activity in the reduction of 4-NP compared to Cu–biochar and N-doped biochar, with complete reduction of 0.35 mM 4-NP within 30 min employing 0.25 g L^−1^ dose. Pseudo-first-order kinetics was observed for 4-NP reduction catalyzed by N–Cu–biochar, and the rate depended on the NaBH_4_ concentration. The findings of this research demonstrated that N-doped biochar with Cu catalysts is a very promising heterogeneous catalyst for 4-NP reduction. The biochar-based catalyst synthesis process can further be incorporated into waste biomass-based energy production processes.^127^

Another study by Bashir *et al.* resulted in the synthesis of a novel nano-catalyst material (nZVI-FBC) from peach skin-derived fibrous biochar and zerovalent iron (nZVI). The catalytic behavior of nZVI-FBC was investigated by the reduction of 4-NP in the presence of NaBH_4._ The nano-catalyst (nZVI-FBC) catalyzes the complete reduction of 4-NP into 4-AP in the presence of NaBH_4._ The time-dependent UV-vis absorption spectrum in Fig. S9 shows decreasing peak intensity at 400 nm (for nitrophenolate) and the emergence of a new peak at 296 nm (for 4-AP), which demonstrates the catalytic reactivity of nZVI-FBC nanoparticles. The nZVI-FBC nanocatalyst exhibits superior catalytic performance for the complete reduction of 4-nitrophenol to 4-aminophenol within 10 minutes. The catalytic reduction process follows pseudo-first-order kinetics, with an activity factor of 25.1 min^−1^ g^−1^. In addition to that, the nZVI-FBC nano-catalyst has high recyclability over numerous catalytic cycles. This catalyst (nZVI-FBC) is extremely promising for the treatment of enormous quantities of wastewater containing toxicants.^128^

Finally, Behera *et al.* prepared Ag@BC-based nanocomposites (Ag@BC-NCs) *via* a two-step process and investigated their catalytic activity toward the reduction of 4-NP. The catalytic efficiency of Ag@BC-NCs was evaluated under various conditions, such as varying NaBH_4_ concentrations, catalysts, Ag-to-biochar ratios, Ag@BC-NC concentrations, pH values, and 4-NP concentrations. The Ag@BC-NCs exhibited excellent catalytic performance for the complete reduction of 4-nitrophenol to 4-aminophenol within 8 minutes. The catalytic reduction process follows first-order kinetics, with a rate constant (*k*_app_) of 4.96 × 10^−1^ min^−1^. Additionally, the reusability of Ag@BC-NCs in the catalytic reduction of 4-NP was examined. The results suggest that the catalytic activity of Ag@BC-NCs remained unchanged throughout the complete reaction and could be recycled for at least three cycles. Thus, these Ag@BC-NCs have the potential to be very promising contenders in the waste biomass sector and render the process economically favourable.^129^Table 4 compares the efficiency of the present catalytic system with the previously reported biochar-based catalysts used for 4-nitrophenol reduction through their *k*_app_. Fig. S10 shows the comparison of the catalytic activity of biochar–metal composites based on their apparent rate constant (*k*_app_) for 4-nitrophenol reduction.

**Table 4:** Comparison of biochar composites and existing catalysts for 4-nitrophenol reduction

Catalyst	Surface area (m^2^ g^−1^)	Pore volume (cm^3^ g^−1^)	Catalyst dosage (mg)	pH	Reduction time (min)	*k*_app_ (×10^− 3^ s^− 1^)	Reusability	Ref.
Ag^0^@CMP	NR	NR	7.4	NR	60	1.40	5	130
Ag@SBA-15	425.00	0.7900	0.9	NR	10	3.40	5	131
Ag NP/Carbon spheres	21.00	NR	1.0	Alkaline condition	25	1.69	NR	132
Fe_3_O_4_@PPy-MAA/Ag	35.69	NR	2.5	NR	45	5.20	8	133
Micron-SiO_2_@nano-Ag particles	NR	NR	10.0	NR	24	2.66	2	134
Ag/Triton X-705	9.26	0.386 × 10^−3^	6.0	NR	30	1.90	NR	135
Ag/PSZN-5	153.39	0.4479	500	NR	3.3	17.29	10	136
Biogenic-Ag nanocatalyst	NR	NR	10	NR	13	1.51	NR	137
AgNP@CCTPB	702.00	1.3900	0.5	NR	3.5	1.20	5	138
Fe3O4@CS_AgNi	321.60	0.3900	2	NR	6	9.33	7	139
Ag@Ca-BC	NR	NR	1	NR	24	4.88	NR	140
SSBC-800	62.29	NR	5	NR	8	8.00	4	9
BC2CuOx	NR	NR	3.5	9.8	0.80	2.33	5	125
N–Cu–biochar	3.80	NR	10	NR	5	NR	NR	127
ZVI-FBC	137.30	NR	10	NR	10	4.20	5	128
AB–NC	3.44	NR	600	NR	8	8.27	8	129
S_1_-CF@PC	61.045	0.060	160	11	10	9.5	5	141
Cu/Biochar	39.400	0.067	4	NR	8	4.5	5	142

#### 4.2.1 Mechanism of reduction

**Fig. 17:**
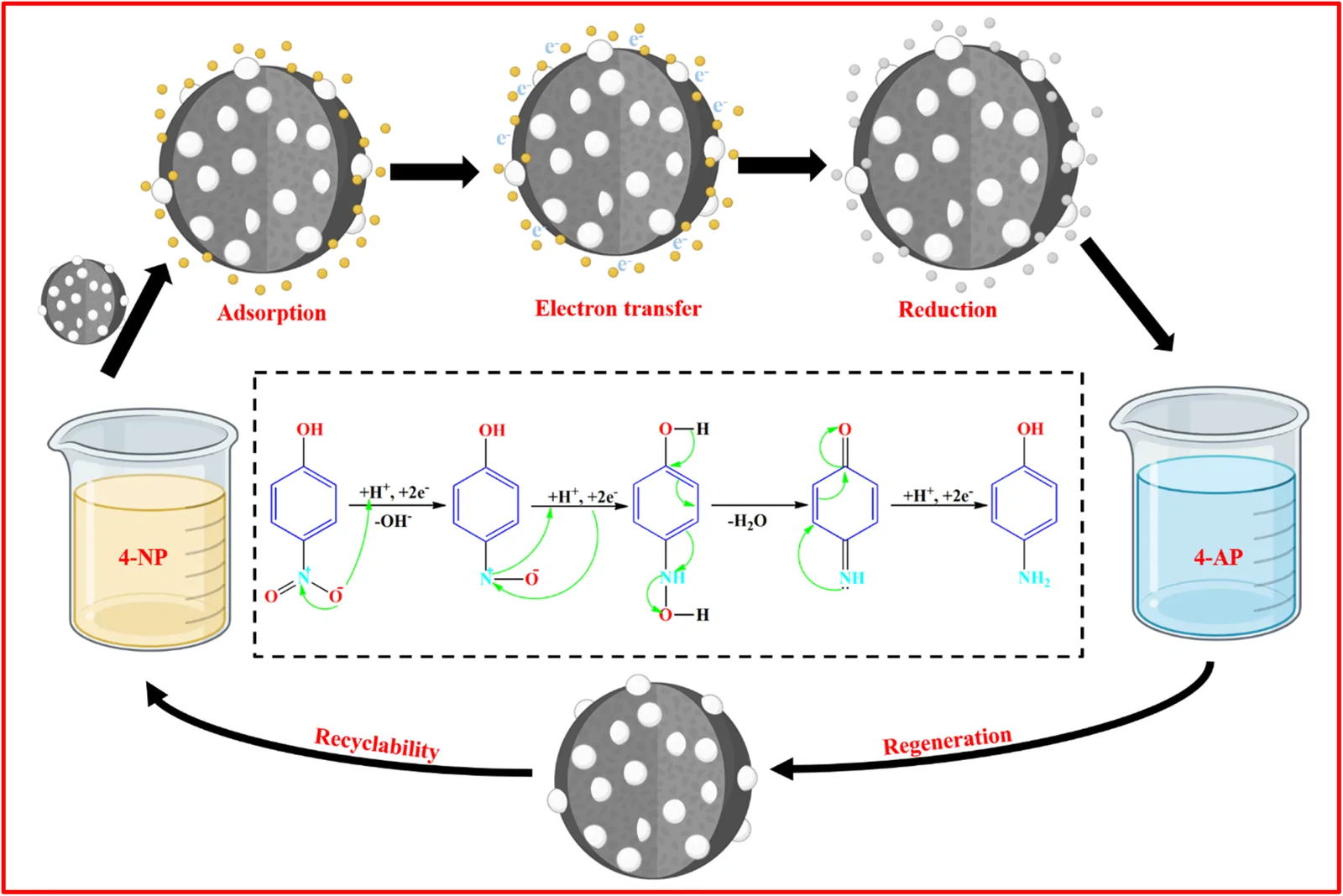
Mechanism of 4-nitrophenol (4-NP) reduction over biochar–metal composites with NaBH_4_.

The mechanism of catalytic reduction of 4-NP over biochar–metal composites can be described in three stages: adsorption of the reactants, electron transfer from borohydride to 4-NP, and desorption of the product. During adsorption, both 4-NP and NaBH_4_ are adsorbed onto the biochar–metal composite surface. The high surface area, well-developed porosity and carbon structure of biochar provide abundant active sites that enhance reactant adsorption, while surface oxygen-containing functional groups (–OH, C–O, C=O, –COO^−^ and –COOH) further increase the adsorption of both the pollutant and the borohydride ion, facilitating the formation of reactive hydride species on the catalyst surface alongside sodium metaborate as a by-product.^140,143,144^ Although NaBH_4_ acts as the hydrogen source and electron donor, direct reduction of 4-NP is kinetically hindered by the high activation barrier between the BH_4_^−^ ion and 4-NP.^145^ The metal nanoparticles dispersed on the biochar surface overcome this barrier by acting as an electron-relay centre, adsorbing both species and transferring electrons and hydride ions from BH_4_^−^ to 4-NP in a six-electron reduction process that converts 4-NP into 4-aminophenol.^146,147^ At this stage, the synergy between the biochar and the metal nanoparticles becomes significant, metal nanoparticles serve as the primary sites for electron transfer, while the biochar provides structural stability and, owing to its semiconducting carbon structure, an additional pathway for electron mobility further lowers the kinetic barrier and accelerates the reaction.^148^ Finally, in the desorption stage, 4-aminophenol desorbs from the catalyst surface, completing the reduction cycle.^149^ The mechanism of 4-nitrophenol reduction over a biochar–metal composite with NaBH_4_ is illustrated in Fig. 17.

## 5 Sustainability and economic aspects

The integration of biochar–metal composites into wastewater treatment systems offers promising opportunities for sustainable development of the systems. To uncover their full potential, it is necessary to carry out detailed evaluations, which include life cycle assessments (LCA), economic feasibility studies and scalability analyses.

### 5.1 Life cycle assessment (LCA)

Life cycle assessment (LCA) is an essential tool for evaluating the environmental implications linked to the production and usage of biochar–metal composites. Through a methodical analysis of each phase, from biomass sourcing and processing to final application, this LCA provides a comprehensive perspective on the environmental footprint, facilitating the discovery of improvement opportunities and assuring adherence to sustainability objectives.^150^

#### 5.1.1 Environmental benefits

Biochar production, especially through pyrolysis of biomass, is currently considered for its role in carbon sequestration and climate change mitigation. The carbon content of biochar is relatively stable, and when applied to soils, it can store carbon for hundreds or even thousands of years, thus lowering the levels of CO_2_ in the atmosphere. Carvalho *et al.* in a study pointed out that incorporating the biochar into soil management could be quite important for climate change mitigation and carbon-efficient resource circulation.^151,152^

Moreover, when biochar is modified with metals to improve its adsorptive capacity, it efficiently removes contaminants including heavy metals and organic pollutants from water. This dual functionality is not only helpful in waste management challenges but also enhances water quality, and thus, contributes to the overall environmental sustainability goals.

#### 5.1.2 Environmental concerns

Despite these benefits, the environmental performance of biochar–metal composites is subject to several constraints. The energy source used during pyrolysis strongly influences the overall environmental impact of the process. For instance, reliance on non-renewable energy can offset the carbon-sequestration benefit. In addition, the potential release of polycyclic aromatic hydrocarbons (PAHs) during pyrolysis poses an environmental and human health risk. A life cycle assessment (LCA) of Fe-modified biochar showed that although the modification enhanced the adsorption efficiency, it also imposed environmental costs associated with iron extraction and wastewater generation during the modification process.^153^

#### 5.1.3 Comparative assessments

LCAs are important tools for comparing the environmental impacts of biochar-based systems with conventional wastewater treatment methods. For instance, a study of the comparison of biochar-amended conservation farming with traditional agriculture in Zambia established that biochar application might result in negative greenhouse gas emissions. However, the authors also noted that the choice of biochar production technology is crucial for the environmental sustainability of the process, and that retort kilns are less harmful than earth-mound kilns.^152,154^

### 5.2 Economic feasibility and scalability

It is important to determine the economic feasibility and scalability for using biochar–metal composites in wastewater treatment applications.

#### 5.2.1 Feedstock availability and raw material cost

One of the most significant economic advantages of biochar–metal composites lies in the use of inexpensive and widely available biomass residues. Agricultural wastes, forestry residues, food-processing by-products, fruit peels, coconut shells, rice husks, corn stalks, sugarcane bagasse, sawdust, sewage sludge, and municipal green waste are generated in enormous quantities worldwide.^155^ These resources often have little commercial value and are frequently disposed of by landfilling or open burning, both of which raise environmental concerns. Converting them into biochars therefore offers value for waste while reducing feedstock purchase costs.^156,157^ Feedstock selection strongly influences overall production economics, since biomass availability, transportation distance, moisture content, ash composition and seasonal variability all affect the processing efficiency. Biomass sources located close to production facilities substantially reduce transportation expenses, whereas centralised processing of geographically dispersed biomass can increase logistics costs and greenhouse gas emissions. Decentralised biochar production units situated near agricultural or forestry operations have therefore been proposed as an economically attractive strategy for reducing transportation costs while improving regional biomass utilisation.^158,159^ Naturally mineral-rich biomass offers a further advantage: feedstocks containing inherent calcium, magnesium, potassium, silicon, or iron require fewer chemical modification steps, reducing reagent consumption and simplifying catalyst preparation.^160^

#### 5.2.2 Production cost of biochar–metal composites

The economic feasibility of biochar-based systems depends on the feedstock, production technology, and operational costs. Using waste biomass as feedstock can lower raw-material costs while contributing to waste management, although the capital cost of pyrolysis equipment and metal-modification processes can be substantial. Among biomass sources requiring waste management, yard waste has been identified as one of the most economically efficient options, particularly when the avoidance of carbon emissions is appropriately priced.^161,162^ Hu *et al.* estimated the production cost of magnetic pomegranate-peel biochar at US$14.3–20.0 kg^−1^, with KOH activation identified as the major cost contributor and biomass feedstock contributing only marginally. The composite retained 76.7% of its adsorption capacity after five regeneration cycles, reducing the effective operating cost to US$2.1–3.3 kg per use, and its magnetic recoverability further reduced separation costs, making it competitive with commercial activated carbon for cost-effective wastewater treatment.^163^

#### 5.2.3 Economic challenges for commercialization

Despite encouraging laboratory-scale results, several economic challenges continue to hinder commercialisation. Variability in biomass composition frequently produces inconsistent biochar properties, requiring additional quality-control measures during manufacturing. The cost of metal precursors, particularly noble metals, remains a significant barrier to large-scale catalyst production. Industrial wastewater streams also contain complex mixtures of inorganic ions, natural organic matter, suspended solids, oils, surfactants, and competing contaminants that may accelerate catalyst deactivation, so catalyst replacement frequency under real operating conditions may differ substantially from laboratory experiments using single model pollutants. Additional uncertainties include regulatory compliance, spent-catalyst waste management, transportation logistics, reactor design optimisation, and integration with existing treatment infrastructure; comprehensive pilot-scale demonstrations are therefore essential before reliable commercial cost estimates can be established.^32,164,165^

#### 5.2.4 Market potential

In addition to wastewater treatment, the market availability of biochar–metal composites can be further developed. Some potential applications are soil enhancement, carbon sequestration, and as additives in construction products. However, market acceptance will require that the performance is consistent, the product is approved by regulators and that it is priced competitively with respect to other solutions available in the market.

#### 5.2.5 Scalability challenges

The overall purpose of this work is to assess the feasibility and challenges of increasing the production of biochar. The consistency of biochar quality and performance is a major challenge because it can be influenced by the properties of biomass feedstock which are variable. This variability can also affect the adsorption capacity and efficiency of biochar. However, there are economic challenges that come with scaling up production such as having to transport biomass to production facilities and then distributing the final product. To address these challenges, decentralized production units close to biomass sources as a potential strategy have been proposed.

#### 5.2.6 Policy and incentives

The government policies and incentives are very important for the economic feasibility of biochar-based technologies. They include subsidies for renewable energy projects, carbon credits for sequestration activities, and grants for sustainable agriculture practices that can cover part of the costs of investment and operation. Additionally, setting up standards and certifications for biochar products, such as those related to quality, safety, and environmental sustainability, can increase consumer confidence and demand in the market.

### 5.3 Regeneration efficiency, catalyst stability and metal leaching

The long-term applicability of biochar–metal composites for wastewater treatment depends not only on their adsorption or catalytic behaviour but also on their ability to retain structural integrity and catalytic activity during repeated use. Although numerous laboratory studies have demonstrated excellent removal efficiencies for organic pollutants, dyes, pharmaceuticals, and heavy metals, catalyst regeneration, stability, and metal leaching remain among the most critical challenges limiting practical implementation. Therefore, systematic assessment of catalyst durability is as important as catalytic efficiency when determining the suitability of biochar–metal composites for industrial wastewater treatment.^71,166^

Unlike homogeneous catalysts, heterogeneous biochar-supported catalysts can be easily separated from treated water and reused for multiple treatment cycles, which significantly reduces operational costs, minimises secondary pollution, and improves the overall sustainability of the treatment process. However, repeated use gradually alters the physicochemical properties of the composite, and these structural changes may reduce the number of accessible active sites, weaken metal–support interactions, and decrease the pollutant removal efficiency.^167^

#### 5.3.1 Regeneration efficiency

Catalyst regeneration refers to the restoration of catalytic activity after each treatment cycle through physical or chemical procedures. Regeneration capability depends largely on the strength of pollutant adsorption, the stability of the metal nanoparticles, and the resistance of the biochar framework to structural degradation. Several regeneration techniques have been reported, including washing with deionized water, ethanol, methanol, dilute hydrochloric acid, or dilute sodium hydroxide, ultrasonic cleaning, and low-temperature thermal treatment. Simple water washing is generally preferred because it minimises chemical consumption and prevents unnecessary degradation of surface functional groups. However, catalysts contaminated with strongly adsorbed aromatic pollutants often require organic solvents or mild thermal treatment to fully restore active sites.^168,169^

Regeneration performance varies considerably among metal systems. Silver-supported biochar catalysts generally show high recyclability owing to the chemical stability of metallic silver under mild conditions. Iron-based composites often maintain high catalytic activity due to strong interactions between iron oxides and oxygen-containing functional groups on the biochar surface. Copper-based catalysts regenerate well under controlled conditions but may undergo gradual oxidation or dissolution in acidic environments. Bimetallic catalysts frequently show high durability owing to synergistic stabilisation between the metallic phases, which reduces nanoparticle aggregation during repeated cycles.^170–172^

Numerous laboratory studies report that biochar-supported catalysts retain a substantial proportion of their initial catalytic activity after multiple reuse cycles. However, considerable variation exists among published studies because regeneration protocols, reaction conditions, pollutant concentrations, and catalyst dosages differ significantly. Future investigations should adopt standardised regeneration procedures and report catalyst performance under identical operating conditions to enable meaningful comparison across catalyst systems.

#### 5.3.2 Catalyst stability during repeated operation

Catalyst stability is one of the most important indicators of long-term performance, since high initial catalytic activity does not guarantee practical applicability. During repeated treatment cycles, deactivation may occur through several mechanisms. Nanoparticle aggregation is a major cause.^173^ Aggregation reduces the number of available active sites for electron transfer, decreasing the catalytic activity. Although the porous structure of biochar restricts nanoparticle mobility to some extent, insufficient anchoring may still permit particle growth after multiple regeneration cycles. Surface fouling is another important pathway, organic intermediates generated during pollutant degradation may accumulate on catalyst surfaces and block active sites, and natural organic matter, suspended solids, oils, surfactants, and inorganic precipitates commonly present in industrial wastewater can cover biochar pores and inhibit reactant diffusion to active metal nanoparticles. Structural degradation of the biochar matrix may also contribute.^174^ Repeated washing, pH fluctuations, oxidative environments and mechanical stirring gradually weaken the carbon structure, damage pore structures and reduce accessible surface area, decreasing the adsorption capacity while weakening interactions between the biochar and the supported metal nanoparticles.^175^ The oxidation of metallic nanoparticles is an additional stability concern, since metallic silver, copper, and zero-valent iron may gradually transform into oxide or hydroxide phases depending on reaction conditions. Although controlled oxidation occasionally enhances the catalytic performance, excessive oxidation generally decreases the electron-transfer efficiency and slows reduction reactions.^176,177^ Maintaining stable oxidation states throughout repeated cycles therefore remains an important objective in catalyst design.

#### 5.3.3 Metal leaching

Metal leaching is widely recognised as one of the key environmental concerns associated with biochar-supported nano-catalysts. During repeated treatment cycles, dissolved metal ions may detach from the catalyst surface and enter the treated effluent, introducing secondary contamination that compromises the environmental sustainability of the process. The extent of leaching depends on numerous factors, including solution pH, ionic strength, reaction temperature, dissolved oxygen concentration, oxidising agents, competing ions, catalyst morphology, nanoparticle size, and metal–support interactions. Acidic conditions generally accelerate the dissolution of transition metals, whereas alkaline environments often improve nanoparticle stability through reduced metal solubility. Therefore, catalyst stability should therefore always be evaluated under realistic wastewater conditions rather than ideal laboratory solutions.^32,173^

Among commonly employed catalytic metals, silver shows relatively low dissolution under neutral conditions but may undergo gradual oxidation in chloride-rich or strongly oxidative environments. Copper generally shows greater susceptibility to dissolution under acidic conditions, while iron-based catalysts may release ferrous or ferric ions through corrosion reactions. Although limited metal release may contribute to homogeneous catalytic activity, excessive leaching reduces catalyst lifetime and raises concerns regarding aquatic toxicity.^178,179^ Reliable quantification of metal leaching requires sensitive analytical techniques capable of measuring trace concentrations of dissolved metals. Inductively coupled plasma-optical emission spectroscopy (ICP-OES) and inductively coupled plasma-mass spectrometry (ICP-MS) have become standard methods for evaluating catalyst stability.^180,181^ Unfortunately, many laboratory studies continue to report catalyst recyclability without measuring dissolved metal concentrations, making it difficult to distinguish true heterogeneous catalysis from homogeneous reactions arising from dissolved metal species.

#### 5.3.4 Strategies for minimizing metal leaching

Several strategies have been developed to suppress nanoparticle leaching while maintaining high catalytic activity. Strengthening the interaction between metal nanoparticles and the biochar surface is the most effective approach. Oxygen-containing functional groups such as hydroxyl, carboxyl, carbonyl, and phenolic groups serve as anchoring sites that improve nanoparticle dispersion and reduce detachment during repeated operation.^182^ Heteroatom doping with nitrogen, sulphur, phosphorus, or boron further enhances metal–support interaction by creating additional coordination sites capable of strongly binding metal nanoparticles. Nitrogen-doped biochar, in particular, has demonstrated an excellent ability to stabilise transition-metal nanoparticles while improving electrical conductivity and electron-transfer kinetics.^183^ Magnetic biochar composites containing iron oxides provide an additional advantage by enabling rapid magnetic separation after treatment, reducing mechanical handling, catalyst loss during recovery, and physical damage over repeated reuse cycles.^184^ Finally, optimising reaction conditions, including pH, oxidant concentration, reducing agent dosage, and catalyst loading, can substantially decrease metal dissolution while maintaining excellent catalytic performance. This operational optimisation is a cost-effective strategy for extending the catalyst lifetime without requiring major changes in the synthesis procedure.

#### 5.3.5 Critical perspective

Although remarkable progress has been made in improving catalyst regeneration and stability, several important knowledge gaps remain. Most published studies evaluate catalyst durability for only a limited number of laboratory reuse cycles under simplified conditions, whereas industrial wastewater treatment systems operate continuously for months or years under highly variable chemical environments. Consequently, stability demonstrated in short-term laboratory experiments may not accurately represent long-term industrial performance. Future research should therefore prioritise long-duration pilot-scale studies, standardised regeneration protocols, comprehensive ICP-OES or ICP-MS monitoring, continuous-flow reactor evaluation, and systematic investigation of catalyst deactivation mechanisms. Regeneration efficiency, metal leaching, catalyst recovery percentage, and structural stability should become mandatory performance indicators reported alongside adsorption capacity and catalytic rate constants. Only through such comprehensive evaluation can biochar–metal composites progress from promising laboratory materials to commercially viable technologies for sustainable wastewater treatment.

## 6 Future research directions

Although significant progress has been made in the development of biochar–metal composites, several scientific and technological challenges that limit their large-scale implementation remain. Future research should focus on improving preparation methods, verifying catalytic mechanisms, establishing long-term stability, and validating practical applicability under realistic operating conditions.

The scalability of preparation methods is a major challenge. Most reported studies have been conducted on a laboratory scale using batch synthesis and reducing agents such as sodium borohydride and hydrazine, which increase the production costs and generate chemical waste. Future work should develop cost-effective, environmentally benign reducing agents, optimise preparation parameters for large-scale production, and establish standardised preparation protocols to improve reproducibility and industrial feasibility.

Most catalytic studies have also been performed under batch conditions using synthetic pollutant solutions, whereas industrial wastewater treatment operates in continuous-flow systems with complex water containing multiple contaminants. Future investigations should therefore evaluate the performance of biochar–metal composites in continuous-flow reactors and pilot-scale systems using real industrial wastewater, providing more realistic assessments of catalyst activity, durability, regeneration efficiency, metal leaching, and operational stability.

A deeper understanding of catalytic mechanisms is also required to guide the rational design of next-generation catalysts. Advanced computational approaches, including density functional theory (DFT), molecular dynamics simulations, and machine learning, should be integrated with experimental investigations to elucidate metal–support interactions, electron-transfer pathways, adsorption energies, and the nature of active catalytic sites. Such approaches can provide atomic-level insights into reaction mechanisms and accelerate the discovery of highly efficient catalyst compositions.

Long-term catalyst stability remains another important research priority. Future studies should systematically investigate deactivation mechanisms such as nanoparticle aggregation, metal leaching, and structural changes during repeated catalytic cycles. Standardised regeneration protocols combined with advanced *in situ* characterisation techniques will provide valuable information on catalyst evolution under reaction conditions and support the development of more durable materials.

Finally, future research should move beyond evaluating the catalytic efficiency alone by incorporating techno-economic analysis (TEA), life cycle assessment (LCA), and environmental risk assessment into catalyst development. Integrating economic feasibility, environmental sustainability, and industrial applicability with catalyst performance will help translate biochar–metal composites from laboratory-scale studies into practical wastewater treatment technologies.

## 7 Conclusion

Biochar–metal composites have lately been recognized as very efficient adsorbents for water treatment, especially for enduring contaminants such as 4-nitrophenol. Combining biochar's advantageous adsorption properties with catalytic metal nanoparticles may enhance efficacy compared to current treatment approaches by decreasing secondary pollutant generation, cutting energy usage and reducing operational expenses. Nonetheless, considerable hurdles remain such as encompassing reliable material performance, economic scalability, environmental repercussions of composite manufacturing and metal leaching. To address these problems, future research must encompass comprehensive mechanistic studies, sophisticated analytical and computational techniques, exploration of novel composite formulations, sustainable synthesis approaches, and life cycle and techno-economic assessments. Extensive testing and approvals are essential for progressing towards practical implementation. Expert assessments from scientists, industry stakeholders, and policymakers underscore the necessity for ongoing research to address the pressing environmental issues confronting society and humanity.

## Author contributions

Abraham Stuvart: writing – original draft, Literature search, Visualization, methodology, formal analysis. Arun Thesingu Rajan: writing – original draft, supervision, literature search, visualization, methodology, project administration, funding acquisition, formal analysis, data curation. Hemanth P K Sudhani: writing – original draft, literature search, visualization, methodology, formal analysis, conceptualization. Natarajan Raman: supervision, resources, investigation, visualization, validation.

## Conflicts of interest

The authors declare no competing interests.

## Supplementary Material

RA-OLF-D6RA05330K-s001

## Data Availability

No primary research results, software or code have been included and no new data were generated or analysed as part of this review. Supplementary information (SI): additional figures that support the discussion presented in this review. See DOI: https://doi.org/10.1039/d6ra05330k.
