# Supplementary material for: Green remediation of 4-nitrophenol-contaminated industrial wastewater using biochar–metal composites: a review
Source: RSC Adv. 2026 Sep 1;16(41):47821–44. doi: 10.1039/d6ra05330k (PMC13531043; doi:10.1039/d6ra05330k)
Supplement: Supplementary file 1 [file RA-016-D6RA05330K-s001.pdf]

Supplementary Information (SI) for RSC Advances.
This journal is © The Royal Society of Chemistry 2026

Supplementary Data

Fig. S1 SEM micrographs of SCG_550 at a magnification of (a) 5 and (b) 25 kX and HZS_550 at (c) 5 and (d) 10 kX

Fig. S2 Morphological analyses of RH and RB

Fig. S3 UV−visible spectra of Ag@biochar and biochar

Fig. S4 FT-IR spectra of pristine biochar and iron-modified biochar

Fig. S5 N_2 adsorption-desorption isotherms of CEB, CEB-AgNP, and CEB-AgNP-673K nanocomposite

Fig. S6 EDX analysis of a) (Bent/AgNPs-Alg) bio-nanocomposite, and b) EDX analysis of MB dye-loaded (Bent/AgNPs-Alg) bio-nanocomposite

Fig. S7 TGA curves of biochar/Fe_3O_4@SiO_2–Ag nanocomposite

Fig. S8 Comparative adsorption capacity of biochar-based composites for 4-nitrophenol removal.

Fig. S9 Time-dependent UV-absorbance spectra of 4-NP reduction using nZVI-FBC nano-catalyst

Fig. S10 Comparison of the catalytic activity of biochar-metal composites based on their apparent rate constants (k_{app}) for 4-nitrophenol reduction

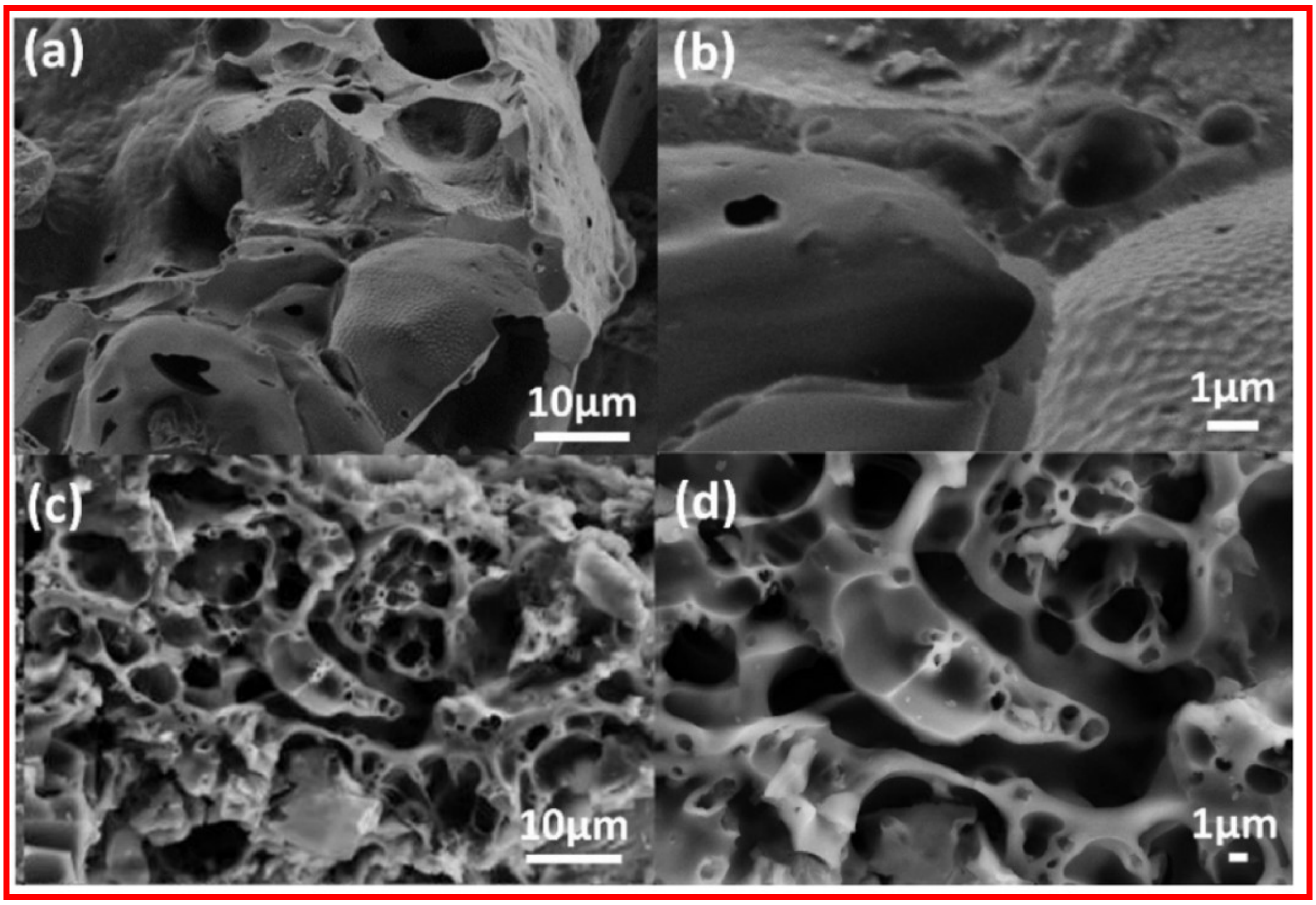

Fig. S1 SEM micrographs of SCG_550 at a magnification of (a) 5 and (b) 25 kX and HZS_550 at (c) 5 and (d) 10 kX [40]

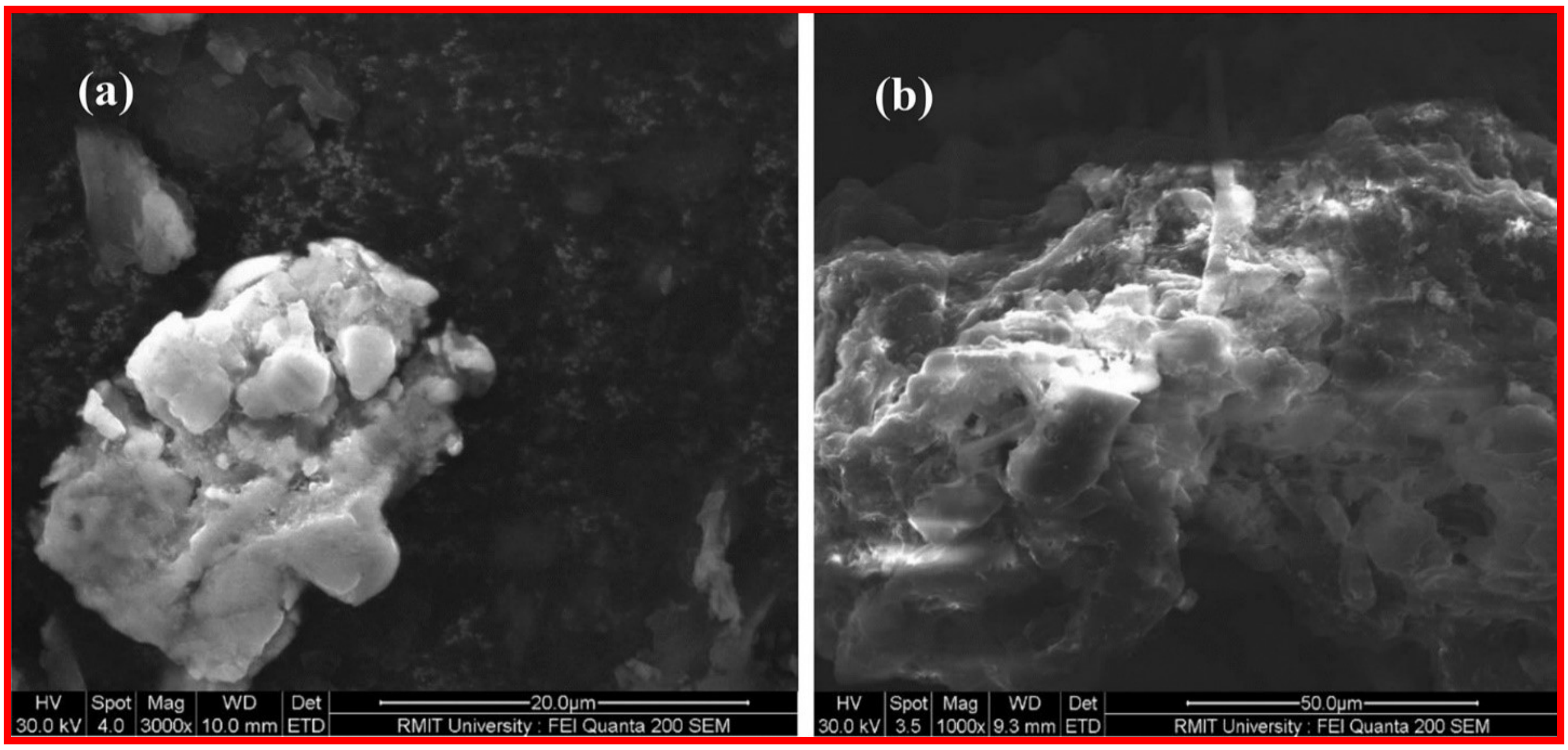

Fig. S2 Morphological analyses of RH and RB[53]

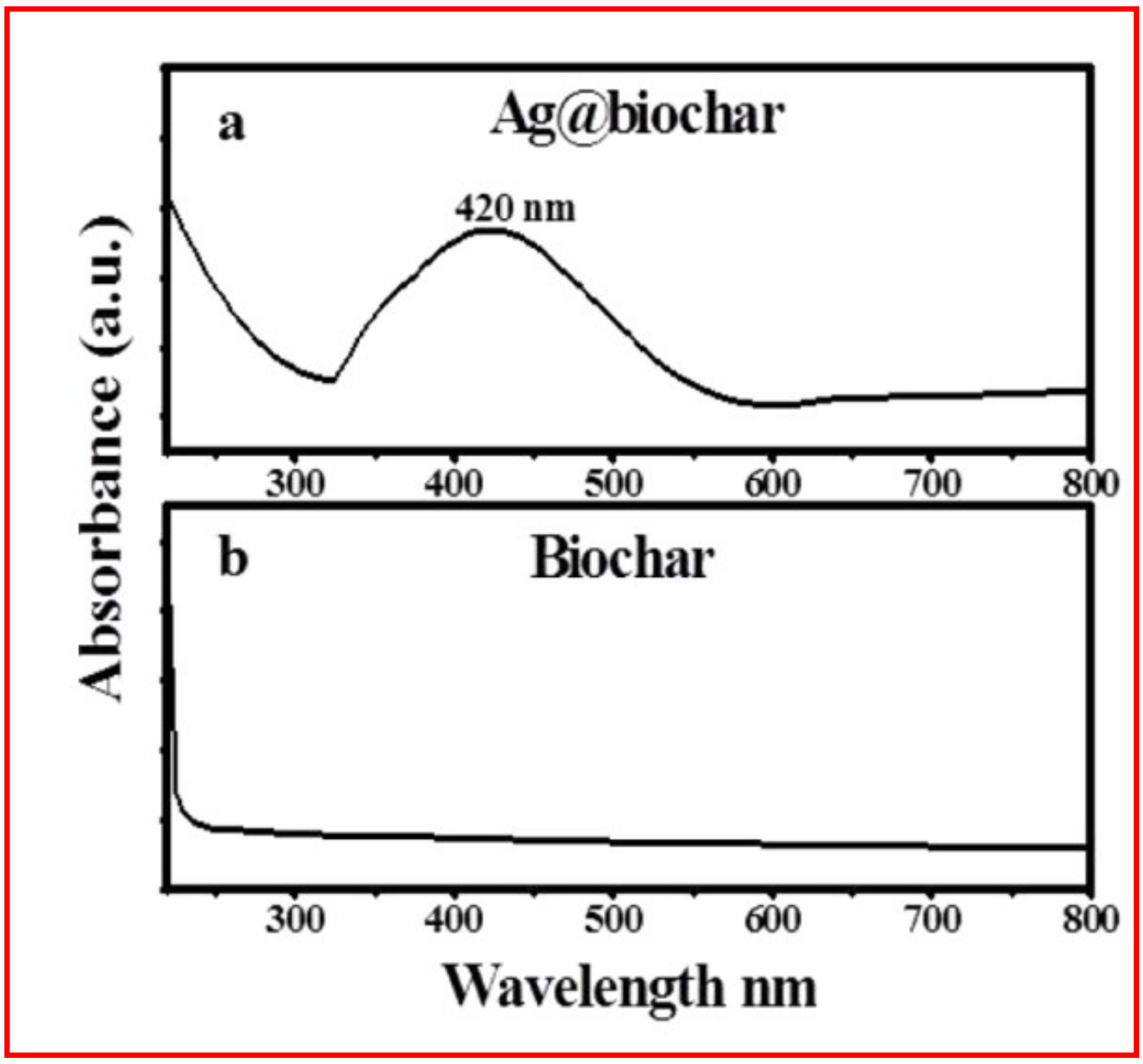

Fig. S3 UV-visible spectra of Ag@biochar and biochar[84]

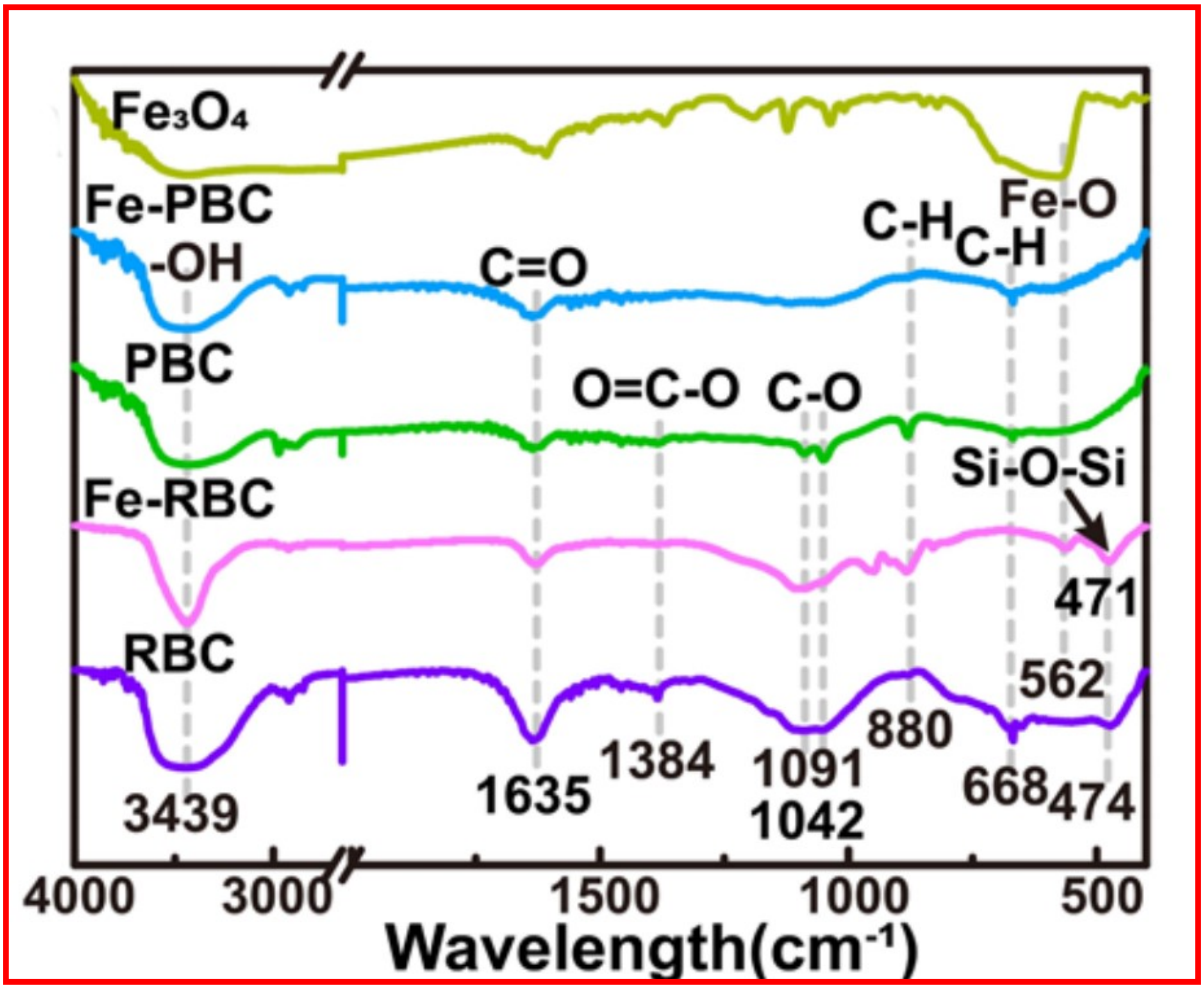

Fig.S4 FT-IR spectra of pristine biochar and iron-modified biochar[87]

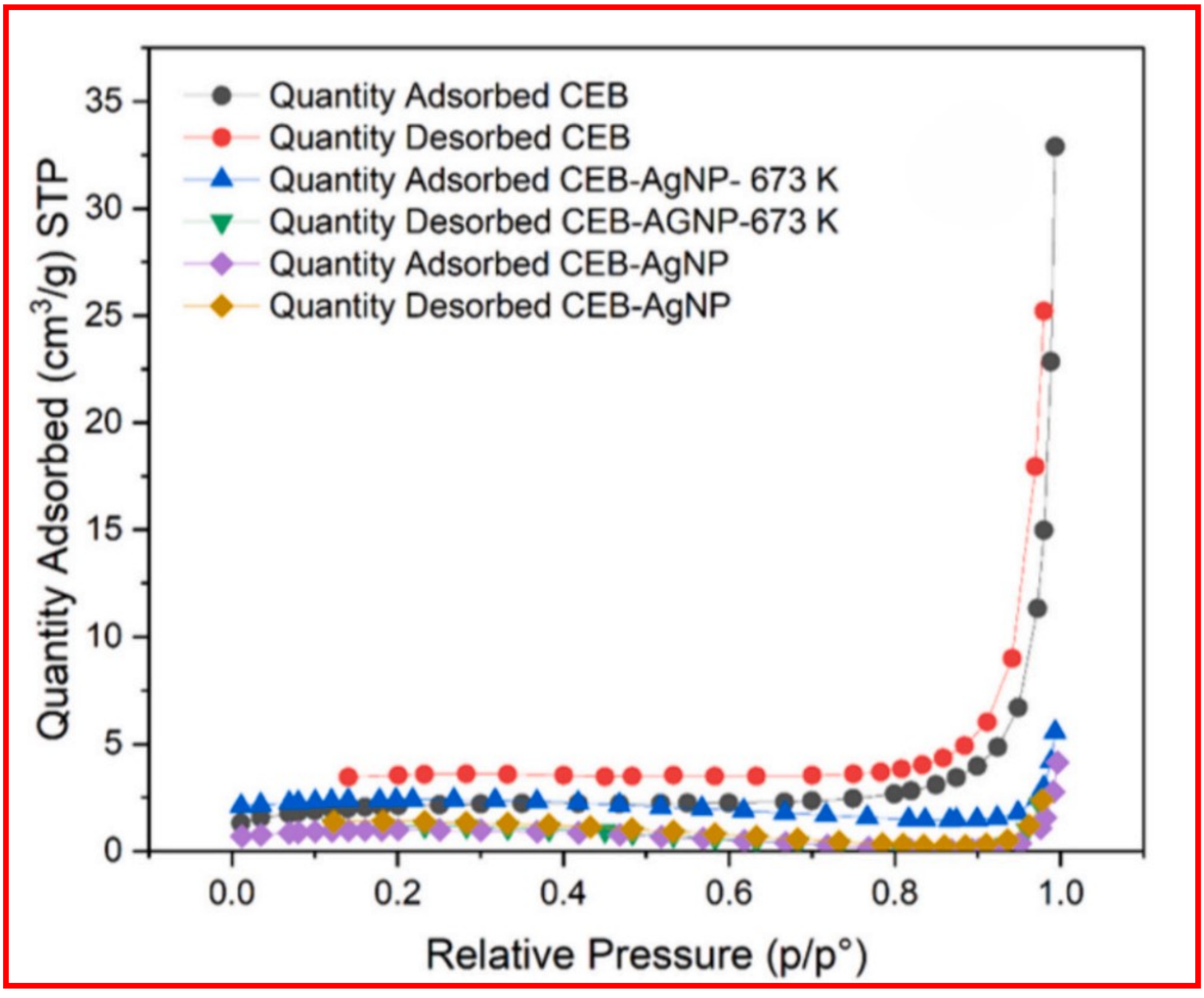

Fig. S5 N_2 adsorption-desorption isotherms of CEB, CEB-AgNP, and CEB-AgNP-673K nanocomposite[90]

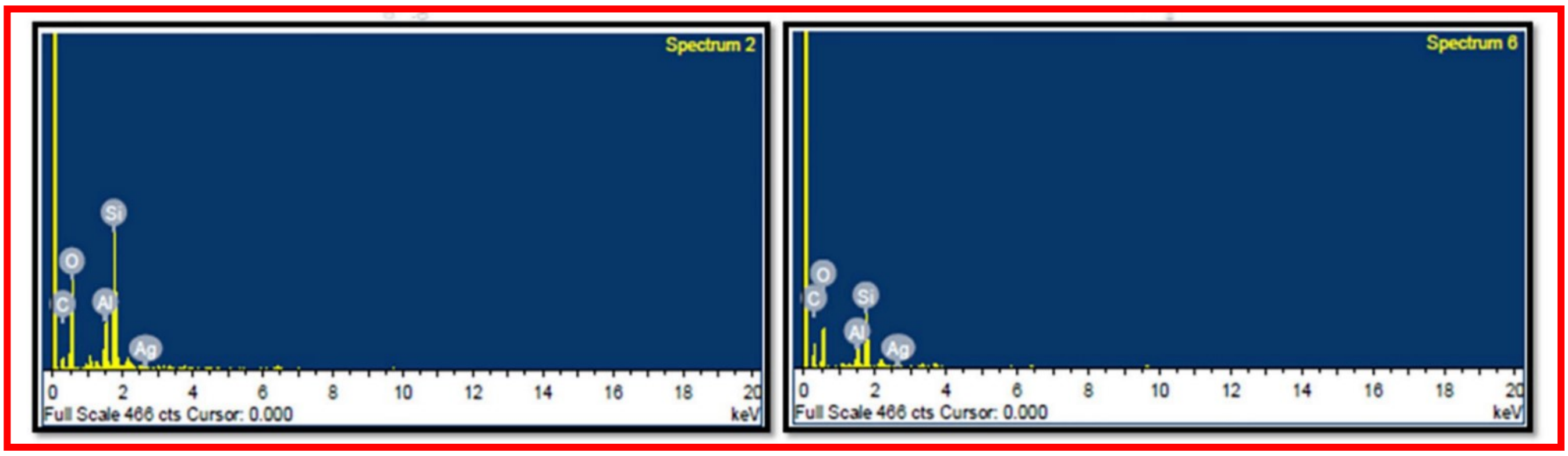

Fig. S6 EDX analysis of a) (Bent/AgNPs-Alg) bio-nanocomposite, and b) EDX analysis of MB dye-loaded (Bent/AgNPs-Alg) bio-nanocomposite[95]

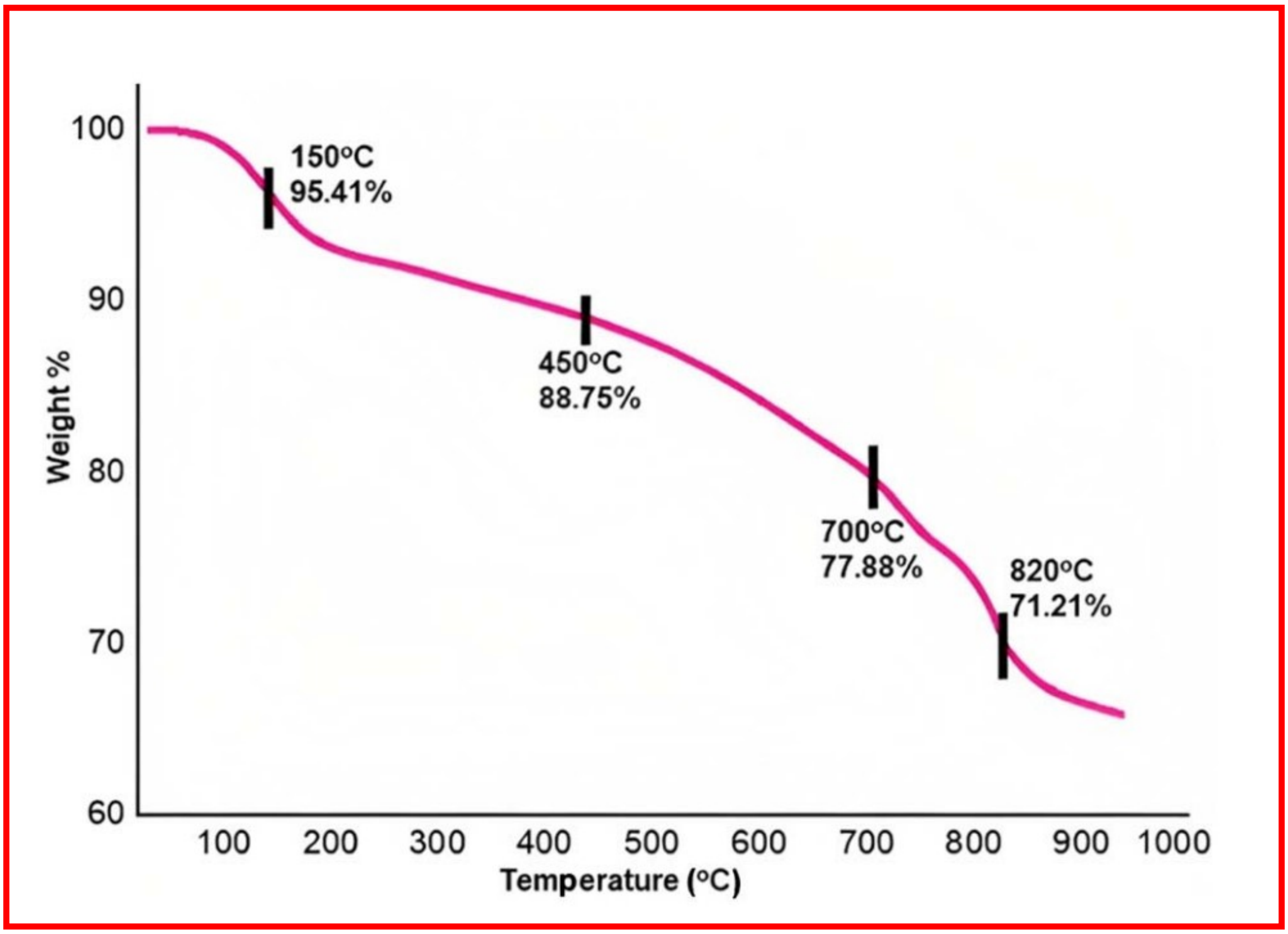

Fig. S7 TGA curves of biochar/Fe_3O_4@SiO_2-Ag nanocomposite[99]

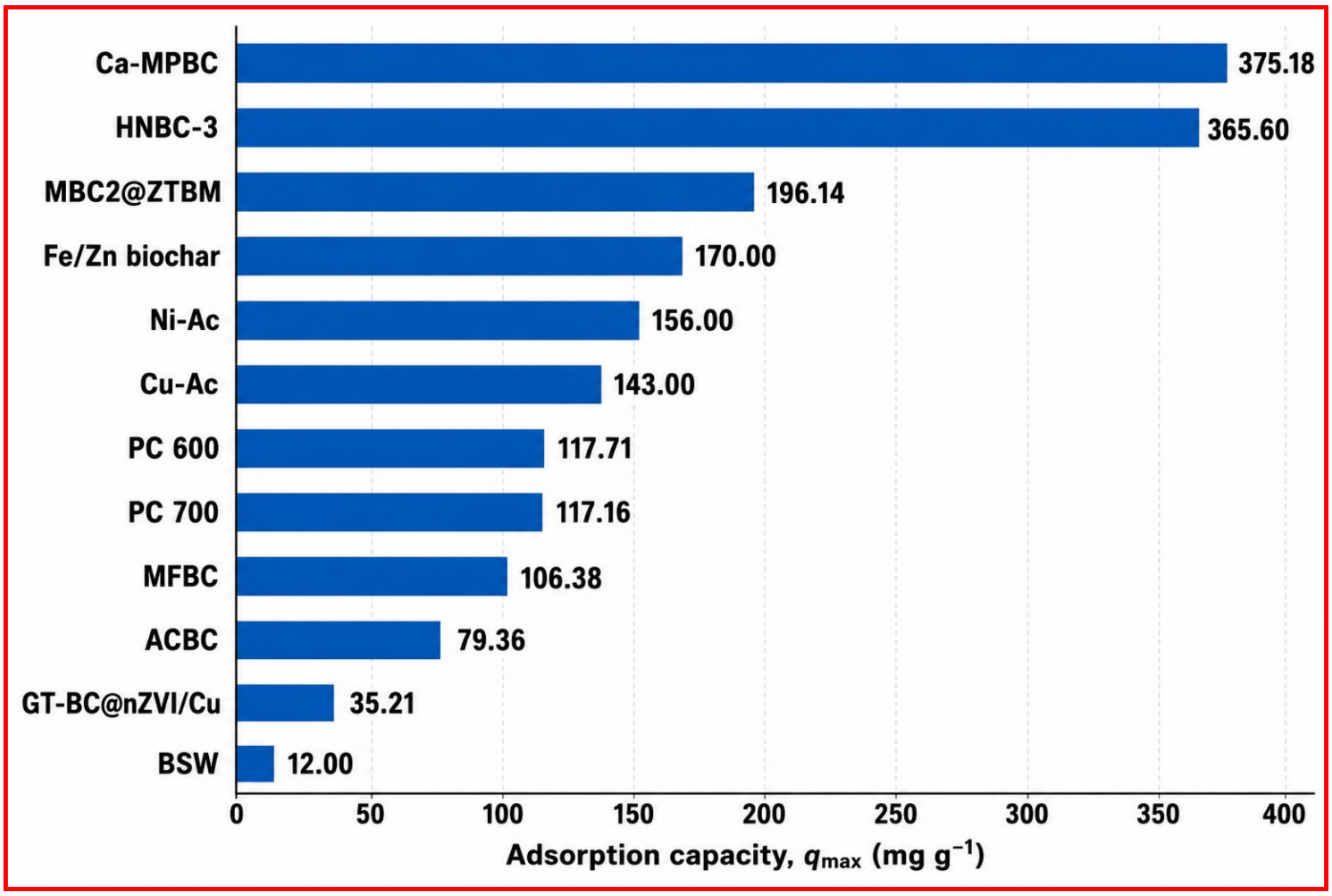

Fig. S8 Comparative adsorption capacity of biochar-based composites for 4-nitrophenol removal.

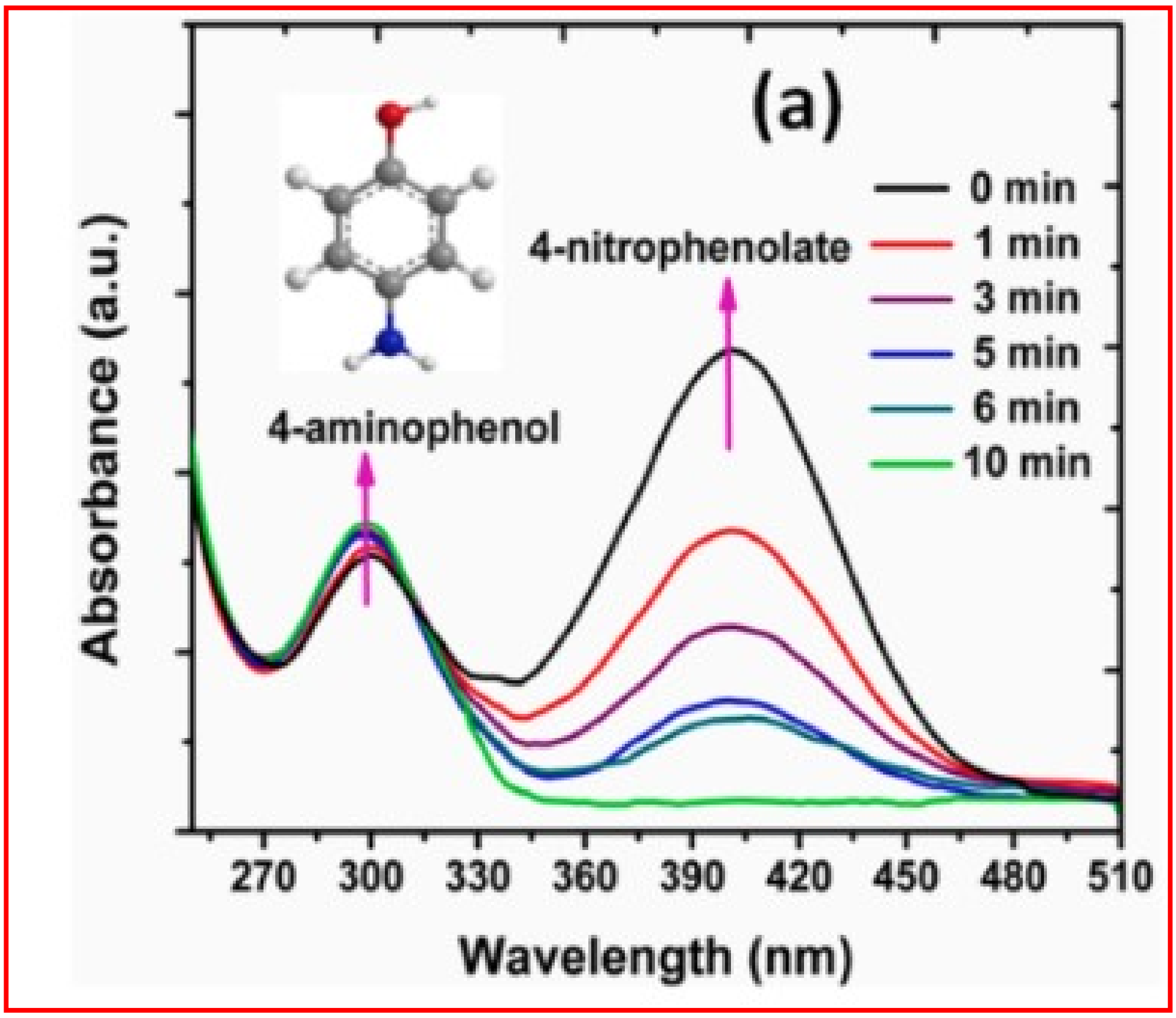

Fig. S9 Time-dependent UV-absorbance spectra of 4-NP reduction using nZVI-FBC nano-catalyst[128]

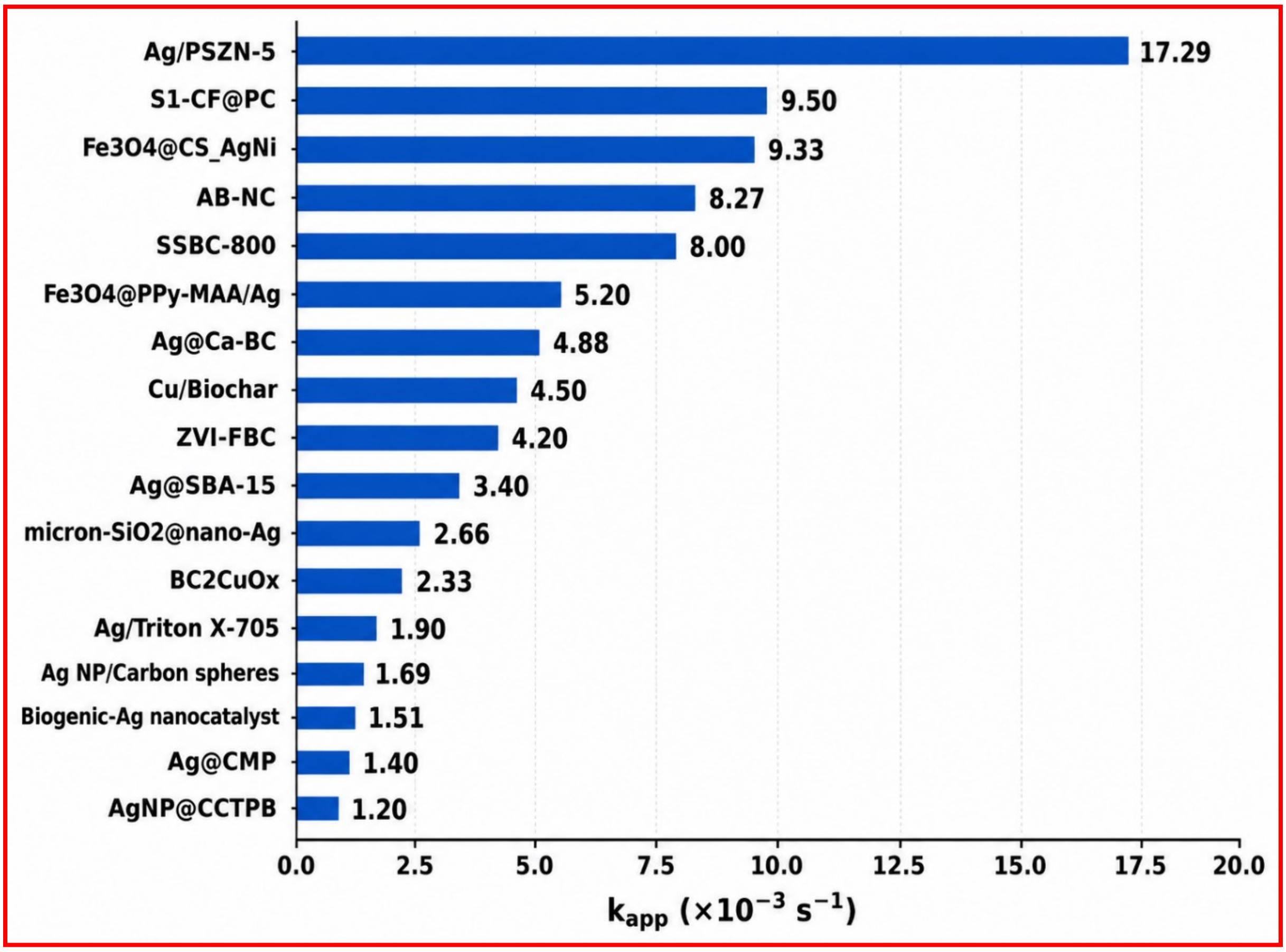

Fig. S10 Comparison of the catalytic activity of biochar-metal composites based on their apparent rate constant (k_{app}) for 4-nitrophenol reduction
